# *LIM Domain Only-2 (LMO2)* Induces T-Cell Leukemia by Two Distinct Pathways

**DOI:** 10.1371/journal.pone.0085883

**Published:** 2014-01-21

**Authors:** Stephen Smith, Rati Tripathi, Charnise Goodings, Susan Cleveland, Elizabeth Mathias, J. Andrew Hardaway, Natalina Elliott, Yajun Yi, Xi Chen, James Downing, Charles Mullighan, Deborah A. Swing, Lino Tessarollo, Liqi Li, Paul Love, Nancy A. Jenkins, Neal G. Copeland, Mary Ann Thompson, Yang Du, Utpal P. Davé

**Affiliations:** 1 Division of Hematology/Oncology, Vanderbilt University Medical Center and the Tennessee Valley Healthcare System, Nashville, Tennessee, United States of America; 2 Department of Pharmacology, Vanderbilt University Medical Center, Nashville, Tennessee, United States of America; 3 Division of Genetic Medicine, Vanderbilt University Medical Center, Nashville, Tennessee, United States of America; 4 Department of Biostatistics, Vanderbilt University Medical Center, Nashville, Tennessee, United States of America; 5 Department of Pathology, St Jude Children's Research Hospital, Memphis, Tennessee, United States of America; 6 Mouse Cancer Genetics Program, National Cancer Institute, Frederick, Maryland, United States of America; 7 National Institute of Child Health and Human Development, National Institutes of Health, Bethesda, Maryland, United States of America; 8 The Methodist Hospital Research Institute, Houston, Texas, United States of America; 9 Department of Pathology, Vanderbilt University Medical Center, Nashville, Tennessee, United States of America; 10 Department of Pediatrics, Uniformed Services University of the Health Sciences, Bethesda, Maryland, United States of America; University of North Carolina at Chapel Hill, United States of America

## Abstract

The *LMO2* oncogene is deregulated in the majority of human T-cell leukemia cases and in most gene therapy-induced T-cell leukemias. We made transgenic mice with enforced expression of *Lmo2* in T-cells by the *CD2* promoter/enhancer. These transgenic mice developed highly penetrant T-ALL by two distinct patterns of gene expression: one in which there was concordant activation of *Lyl1*, *Hhex*, and *Mycn* or alternatively, with *Notch1* target gene activation. Most strikingly, this gene expression clustering was conserved in human Early T-cell Precursor ALL (ETP-ALL), where *LMO2*, *HHEX*, *LYL1*, and *MYCN* were most highly expressed. We discovered that *HHEX* is a direct transcriptional target of *LMO2* consistent with its concordant gene expression. Furthermore, conditional inactivation of *Hhex* in *CD2-Lmo2* transgenic mice markedly attenuated T-ALL development, demonstrating that *Hhex* is a crucial mediator of *Lmo2*'s oncogenic function. The *CD2-Lmo2* transgenic mice offer mechanistic insight into concordant oncogene expression and provide a model for the highly treatment-resistant ETP-ALL subtype.

## Introduction

Mammalian genomes have 4 *LIM domain Only* paralogs that are causally implicated in several human cancers such as T-cell leukemia (*LMO1/2*), neuroblastoma (*LMO3*), lung cancer (*LMO3*), and breast cancer (*LMO4*)[1], [2]. *LMO1/2* are transcriptionally deregulated in the majority of human acute T-cell lymphoblastic leukemia (T-ALL) patients[3]. *LMO2* was originally identified from recurrent chromosomal translocations involving T-cell receptor genes whose regulatory elements were positioned 5′ of the first exon of *LMO2*. More recently, *LMO2* deregulation has been attributed to interstitial deletions and other chromosomal rearrangements [4], [5]. *LMO2* was insertionally mutated by gammaretroviral gene therapy vectors in X-linked severe combined immunodeficiency (SCID-X1) and Wiskott-Aldrich syndrome [6]–[9]. The gene therapy vectors integrated 5′ of *LMO2* coding sequences, induced overexpression and triggered T-ALL 2–3 years after retroviral transduction. Hence, deregulated *LMO2* expression is an early mutational event in T-ALL. This is demonstrated in mouse models like bone marrow chimeras and transgenic mice where *Lmo2* expression is enforced from constitutive promoters[3],[10]. We identified *Lmo2* as a frequent integration site in AKXD mice where retroviral integration analysis and gene expression proved to be informative in modeling gene therapy-induced T-ALLs [11], [12].

The gene therapy experience and mouse models show that *LMO2* expression can be enforced in hematopoietic stem and progenitor cells (HSPCs) but only T-cell progenitors are clonally selected and transformed [6]. The earliest T-cell progenitor cells express *Lmo2* but expression is down-regulated in developing T cells and completely repressed in mature T cells[13]. *Lmo2* overexpression in T-cell progenitors caused differentiation block, quiescence, and increased self-renewal [14]–[16]. These are all hallmarks of HSCs and indeed *Lmo2*-expressing T-cell progenitors show an HSC-like transcriptional profile[16]. We speculated that *Lmo2* may be a driver of these HSC-like features since *Lmo2* is required for the specification of normal adult and primitive HSCs. *Lmo2^-/-^* ES cells contribute to diverse tissues in blastocyst chimeras but not to hematopoiesis[17]. However, *Lmo2* conditional knockouts show that it is not necessary for T- or B-cell development [18].

In normal erythroid progenitor cells, Lmo2 is part of a large macromolecular complex comprised of Tal1/Scl (a class II basic helix-loop-helix transcription factor), Gata1, E47 (a class I bHLH protein), LIM domain binding 1(Ldb1), and Single-stranded DNA binding protein 2 (Ssbp2)[19], [20]. This protein complex assembles at E box-GATA sites in erythroid target genes. The nature of this complex in HSCs has not been well characterized but Gata2 and Lyl1 may substitute for Gata1 and Tal1, respectively. Germline deletion of these proteins causes loss of primitive hematopoiesis and induces embryonic lethality at the same approximate developmental stage, underscoring the importance of the complex in HSC maintenance [21].

It is likely that LMO2 and its protein partners in normal HSPCs also associate in T-ALL because many of them are co-expressed in the leukemias. Gene expression analysis of human and murine T-ALL show concordant expression of *LMO2* and bHLH genes, *TAL1*, *LYL1*, and *OLIG2* [7], [12], [22]. Here, we analyzed the gene expression of T-ALLs developing in *CD2-Lmo2* transgenic mice. We found that *Lyl1* was the predominant bHLH upregulated in the majority of T-ALLs. The gene expression of this model and human T-ALL showed two distinct mutually exclusive transcriptional profiles. *Lmo2* and *Lyl1* were concordantly expressed in a profile that included *Hematopoietically expressed homeobox* (*Hhex*) and *Mycn* genes. These same genes are highly expressed in Early T-cell Progenitor ALL, a treatment-resistant T-ALL subtype. We discovered that *Hhex* is a direct transcriptional target of *Lmo2* and a crucial mediator of the oncogenic functions of *Lmo*2.

## Results

### *CD2-Lmo2* transgenic mice develop highly penetrant T-ALL with upregulation of *Lyl1*

**Figure 1:**
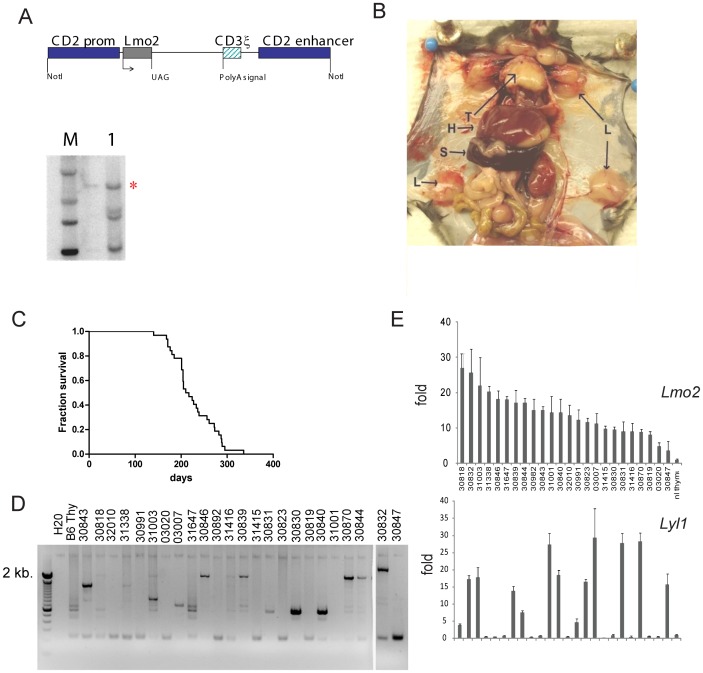
*CD2-Lmo2* transgenic mice develop T-ALL with high penetrance, long latency and upregulation of *Lyl1*. (**A**) schematic shows structure of *CD2-Lmo2* transgene used in pronuclear injection. Lower panel shows a Southern blot of tail genomic DNA digested with *Eco*RI which cuts once within the transgene and probed with *Lmo2* cDNA. Red asterisk shows transgene band in equal intensity to other bands which represent the endogenous *Lmo2* exons. (**B**) photo of *CD2-Lmo2* transgenic mouse sacrificed due to T-ALL onset. T, thymus; H, hepatomegaly; L, lymphadenopathy; S, splenomegaly. (**C**) Survival analysis of *CD2-Lmo2* transgenic mice (n = 24). (**D**) PCR analysis of *Jβ2* T-cell receptor showed rearrangement in most T-ALL genomic DNA; germline configuration is shown by the presence of only a 2 kb band in the lane. (**E**) *Lmo2* and *Lyl1* mRNA were quantified by qRT-PCR on whole RNA isolated from 24 *CD2-Lmo2* T-ALLs and shown relative to levels in normal thymus. Values shown are fold increase over normal thymus.

We cloned the mouse *Lmo2* cDNA into the human *CD2* promoter/enhancer construct (Figure 1A)[23] and created transgenic mice in B6C3HF2 hybrids; these mice were then backcrossed to B6 mice. We have previously shown that these transgenic mice have enforced expression of *Lmo2* at the double negative stage of T-cell development where no endogenous *Lmo2* is detectable [16]. T-cell acute lymphoblastic leukemia (T-ALL) presented with massive organomegaly and bone marrow involvement (Figure 1B) at a median latency of 230 days and 100% penetrance (Figure 1C) similar to what has been described in independently constructed *CD2-Lmo2* transgenics; the latency was increased with backcrosses to B6 (Figure S5 in File S1) [24], [25]. Twenty out of 24 (83%) T-ALLs showed clonal *Jβ2* T-cell receptor rearrangement; the rest had germline configurations (Figure 1D). *Lmo2* mRNA was increased in the transgenic T-ALLs at levels 5-10 fold above normal thymus as analyzed by qRT-PCR (Figure 1E). In human T-ALLs, *LMO2* or *LMO1* overexpression overlaps with overexpression of class II bHLH genes: *TAL1*, *LYL1*, or *OLIG2* so we analyzed these in the transgenic T-ALLs by qRT-PCR. *Lyl1* was the only class II bHLH gene overexpressed in 54% (13/24) of the transgenic T-ALLs (Figure 1E). Only 1 out of 30 T-ALLs showed upregulation of *Tal1* and no tumors expressed *Olig2* or *Tal2* (data not shown). This was markedly different from our results on AKXD T-ALLs initiated by retroviral insertional activation of *Lmo2* which showed upregulation of *Lmo2* and *Tal1* mRNA [26]. The *CD2-Lmo2* transgenics resembled human *LYL1*-overexpressing T-ALLs which are similarly concordant with *LMO2* overexpression and not with other *LMO* paralogs [22].

### LMO2, LYL1, and LDB1 are part of a protein complex in T-ALL cells that binds tandem E boxes

**Figure 2:**
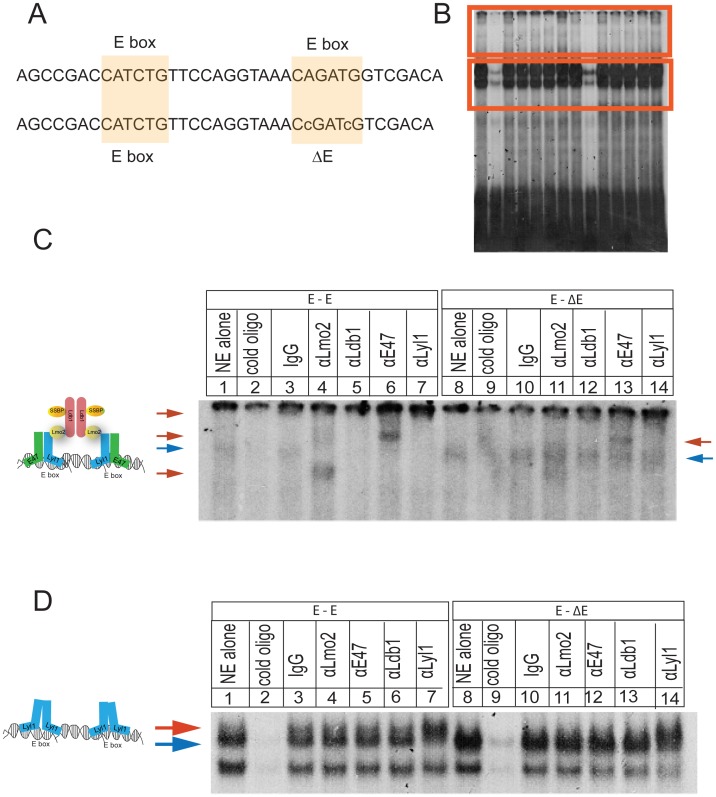
LYL1 is part of an LMO2-associated protein complex in T-ALL cells that binds tandem E boxes. (**A**) shows oligos used in EMSA. First oligo has two E boxes separated by 11 bp; second oligo has a scrambled second E box. (**B**) Oligos were end-labeled with ^32^P-ATP and used to bind nuclear extract from LOUCY cells, a T-ALL line that expresses *LMO2* and *LYL1*. The gel shows migration of two different complexes, boxed in red, a slow and fast complex. (**C**) The slow complex was close to the origin and was resolved with an overnight run on 4% polyacrylamide gel. Blue arrows show the macromolecular complex that is competed with cold oligo and shifted in the presence of antibodies to LMO2, LDB1, E47, and LYL1. The red arrows show altered mobility of protein complexes. (**D**) shows the faster migrating complex (blue arrow) that is competed away by cold oligo and supershifted with antibody against LYL1 (red arrow).

Next, we investigated the functional significance of *Lyl1* upregulation in *Lmo2*-induced T-ALL. In normal HSCs and erythroid progenitors, Lmo2 protein binds to bHLH protein Tal1, Gata1 or Gata2, and LIM domain binding protein 1 (Ldb1), and Ssbp2 to regulate transcription of genes with E box-Gata sequences within their regulatory sequences [20]. In T-ALL, Lmo2 and Ldb1 appeared to bridge two Tal1/E47 heterodimers that were bound to tandem E boxes [27]. To test whether LYL1 is part of a similar complex, we used electrophoretic mobility shift assay (EMSA). We could not isolate nuclear extracts from the murine T-ALLs that were suitable for EMSA and therefore prepared nuclear extract from a human T-ALL cell line, LOUCY, which resembles our transgenic mouse tumors (i.e. overexpresses *LYL1* and *LMO2* but not *TAL1*). We observed two major species of complexes that bound ^32^P-labeled oligo with tandem E-E boxes: a faster migrating complex (Figure 2B, lower red box) and a slower, high molecular weight complex closer to the origin (Figure 2B, upper red box) that entered the gel after a much longer run when free probe was run off. These complexes were analyzed on separate gels and both were competed with cold oligo (Figure 2C–D, lanes 2 and 9).

The faster migrating complex shown in Figure 2D, appeared as a doublet for the E-E sequence but as a single band for E-ΔE (compare band shown by blue arrow in lanes 1 and 8 in Figure 2D). This complex was supershifted (red arrow) from both sequences with only anti-LYL1 antibody (see lane 7 and 14, Figure 2D) suggesting that this was the only protein bound to the E-boxes, perhaps as a homodimer [28]. There was a different pattern observed for the high molecular weight complex. The mobility of this complex (blue arrow, Figure 2C) did not change with the addition of nonspecific IgG (Figure 2B, lane 3) but was disrupted (red arrows) with antibodies against LMO2, LYL1, E47, and LDB1 (Figure 2C, lanes 4–7) or supershifted in some cases to the origin. A complex of similar size bound the E- ΔE oligo (Figure 2C, lanes 1 and 8) but it was partially shifted with anti-E47 only. Interestingly, the presence of anti-LMO2 caused a supershift and a faster migrating complex for E-E oligo suggesting disruption of the macromolecular complex or a change in the conformation of the complex (Figure 2C, lane 4). Significant supershifts or disruptions of the complex were only observed for the tandem E box oligos showing that LYL1, E47, LMO2, and LDB1 are part of a common macromolecular complex in T-ALL cells.

### Gene expression analysis of oncogenes reveals two distinct classes of *CD2-Lmo2* T-ALLs

The long latency of T-ALL onset in *CD2-Lmo2* transgenic mice implied that other mutations in cooperating oncogenes are required. We had previously identified cooperating oncogenes in the AKXD murine model based on concordant integrations with *Lmo2* [12]. We analyzed the *CD2-Lmo2* T-ALLs to see if these same oncogenes were overexpressed. Similar to our analysis for bHLH gene expression, the *CD2-Lmo2* T-ALLs were different from most of the AKXD T-ALLs and did not overexpress the same oncogenes (data not shown). For example, *Mef2c* was expressed in only 1 out of 30 *CD2-Lmo2* T-ALLs analyzed by qRT-PCR (data not shown), interestingly, in the same T-ALL that displayed *Tal1* upregulation. This suggested to us that those oncogenes cooperating with *Lmo2* in the context of *Tal1* upregulation may not be the same in the context of *Lyl1* upregulation. We discovered that *Lyl1* and *Hhex* were concordantly expressed in pre-leukemic *CD2-Lmo2* transgenic T-cell progenitors that also had an HSC-like gene expression signature, differentiation arrest, quiescence, and increased self-renewal, which is consistent with findings from independently engineered transgenic mice[16], [29].

**Figure 3:**
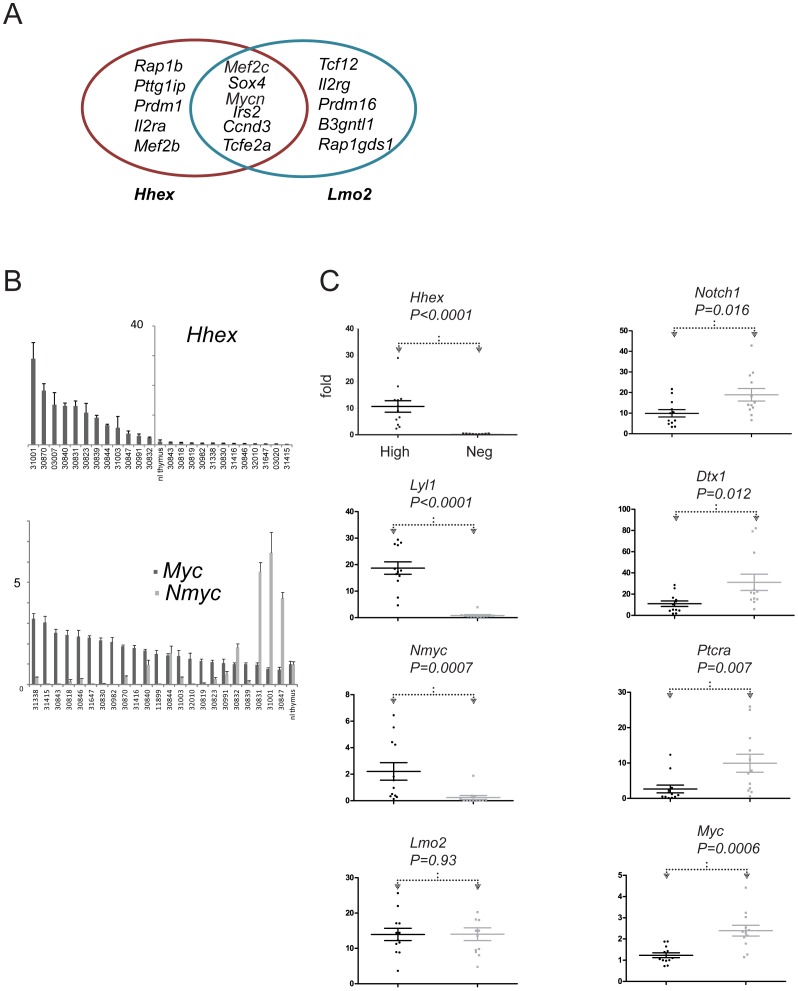
*Hhex* expression defines two subtypes of *CD2-Lmo2* transgenic T-ALLs. (**A**) We analyzed the Retroviral Tagged Cancer Gene Database (RTCGD) for T-ALLs with integrations at *Lmo2* and at *Hhex*. These two genes were never mutated in the same tumor but we observed overexpression of *Hhex* in all the *Lmo2*-clonal T-ALLs [12]. The red set (left) of genes were all insertionally mutated concordant with *Hhex* whereas the blue set (right) of genes was concordant with *Lmo2*. The genes shown in the overlap, *Mef2c*, *Sox4*, *Mycn*, *Irs2*, and *Ccnd3*, and *Tcfe2a*, were concordant with both *Hhex* and *Lmo2*-induced T-ALLs. (**B**) Bar graph shows qRT-PCR analysis for *Hhex* transcripts in whole RNA isolated from *CD2-Lmo2* T-ALLs. All values are expressed as fold increase above normal thymus and are shown from highest to lowest relative expression. Tumor names appear on x axis. The y-axis was placed between those T-ALLs that expressed *Hhex* and those that did not. Bar graph shows similar quantification of *Mycn* and *Myc*. Here, the T-ALLs are arranged from highest to lowest relative expression of *Myc* and values are shown as fold increase over normal thymus. The dark gray bars show *Myc* and light gray show *Mycn* mRNAs. (**C**) We divided *CD2-Lmo2* transgenic T-ALLs into two classes, those with high *Hhex* expression (left of y axis in panel A) and those with negative or low *Hhex* expression (right of y axis in panel A) and plotted the relative expression values for the genes shown. The mean values of the genes are shown by the horizontal bars. The mean expression for each gene was compared by two tailed Mann-Whitney U-test generating the P values shown.

*Hhex* was notable for being upregulated in the AKXD T-ALLs without insertional mutation despite the fact that it is the second most common integration site in the Retroviral Tagged Cancer Gene Database (RTCGD). We analyzed all AKXD T-ALLs with integration at *Hhex* and *Lmo2* for concordant integrations. *Hhex* integrations were not concordant with *Lmo2* but there were six genes, *Mef2c*, *Mycn*, *Irs2*, *Ccnd2*, and *Tcfe2a* that were concordant with *Lmo2* and with *Hhex* in independent T-ALLs (Figure 3A). This remarkable degree of overlap in concordant integrations suggested to us that *Hhex* and *Lmo2* operate in the same oncogenic pathway analogous to genetic epistasis. We observed *Hhex* expression in 12/24 (50%) transgenic T-ALLs by qRT-PCR (Figure 3B). We hypothesized that the comparison between *Hhex*-high and *Hhex*-negative T-ALLs may be biologically meaningful so we divided the murine T-ALLs into two classes. Remarkably, *Hhex* (*P* = .0001), *Lyl1* (P<.0001), and *Mycn* (*P* = .007) clustered with the *Hhex*-high class (Figure 3C).

Interestingly, the tumors in which *Mycn* was upregulated showed the lowest levels of *Myc* expression and *Myc* was significantly upregulated in the *Hhex*-negative T-ALLs (Figure 3B–C). Since *Myc* is transcriptionally activated by cleaved Notch1 protein, we analyzed other Notch target genes such as *Notch1*, *Dtx1*, *Ptcra*, and *Hes1* [30]–[32]. All these genes except *Hes1* (*P* = .406) were expressed at higher levels in the *Hhex*-negative T-ALLs compared to the *Hhex*-high T-ALLs (Figure 3C). We found mutations in 100% of the T-ALLs which were mostly insertions in exon 34 that caused frameshifts amino terminal to the PEST degradation sequence (Table S3 in File S1)[33]. The same types of mutations were present in *Hhex*-high and *Hhex*-low tumors even though the mRNAs of *Dtx1*, *Notch1*, and *Myc* varied significantly. Survival analysis showed that *Hhex*-high tumors developed T-ALL with longer latency than *Hhex*-low T-ALLs; and, *Dtx1*-high T-ALLs developed at a shorter latency than *Dtx1*-low expression (Figure S4 in File S1). In summary, *Hhex* expression and *Notch1* target gene expression defined two mutually exclusive gene expression patterns in *CD2-Lmo2* transgenic T-ALLs.

### Oncogenes overexpressed in *CD2-Lmo2* transgenic T-ALLs are also deregulated in human T-ALL

**Figure 4:**
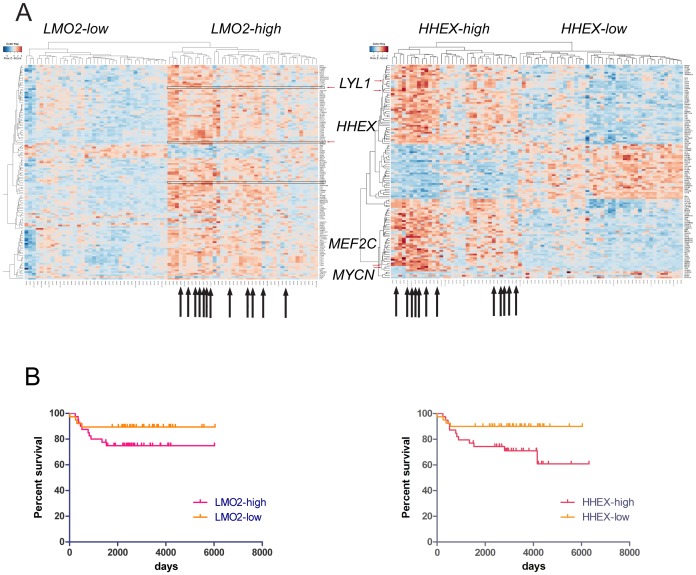
*LMO2* and *HHEX* are co-expressed in ETP-ALL. (**A**) The heat map shows a supervised clustering result based on *LMO2* (left panel) and *HHEX* (right panel) expression. The colors denote z scores ranging from −4 to 4. The gene expression dataset of 78 pediatric T-ALLs was analyzed by *Limma* based on a discrete cutoff of median *LMO2* and *HHEX* expression. The black arrows show 12 Early T-cell Precursor ALL cases. *LYL1*, *HHEX*, *MEF2C*, and *MYCN* genes were concordantly expressed with *LMO2* and *HHEX*. These genes were expressed highest in the ETP-ALL cases. A full version of the heat maps are in file S1. (B) Survival analysis was performed on LMO2-high versus LMO2-low T-ALL and on HHEX-high v. HHEX-low T-ALLs. The curves for *LMO2* classes in the left panel were not statistically significant (*P = .19*) but the curves for *HHEX* classes were significantly different (*P = .03*) by Log rank tests.

To validate the oncogenic signatures we observed in *CD2-Lmo2* transgenic T-ALLs, we analyzed 78 pediatric cases by supervised clustering based on *LMO2* expression. Forty pediatric T-ALL cases expressed *LMO2*; 39 T-ALL cases expressed *HHEX*; 6 cases expressed *LMO2* without *HHEX* and 6 cases expressed *HHEX* without *LMO2*; *LMO2* and *HHEX* were concordantly expressed in 34 cases which were statistically enriched for *LYL1*, *MEF2C*, and *MYCN*. These oncogenes were most highly expressed in all 12 cases of Early T-cell Precursor ALL (ETP-ALL) included in this cohort which also clustered together in the *LMO2*-high group of T-ALLs (see arrows, Figures 4A and S1 in File S1). *LYL1* was expressed at higher levels in the Early T-cell Precursor subtype of T-ALL [15], [22], [34]. Therefore, we repeated the supervised clustering of pediatric T-ALL based on *HHEX* expression and again, all 12 ETP-ALLs clustered together, a statistically significant result (see black arrows in Figures 4A and S2, Fisher exact test, *P* = .0002). The *HHEX*-positive T-ALLs showed statistically significant, concordant expression of *LYL1*, *MYCN*, *LMO2*, *BCL2*, and *MEF2C*. We confirmed the concordant expression of *HHEX* and *LMO2* by qRT-PCR analysis on an independent set of 23 primary T-ALL samples (Figure S3 in File S1). The *CD2-Lmo2* transgenic T-ALLs overexpressing *Hhex* resembled the ETP-ALL subtype in their expression of *Lyl1* and *Mycn*. ETP-ALLs showed a much lower rate of *NOTCH1* gene mutation compared to non-ETP cases, analogous to decreased *Notch1* target gene activation in the murine *Hhex*-negative T-ALLs. NOTCH1 targets, *MYC* (FDR = .0355), *PTCRA* (FDR = 4.2×10^−9^), and *NOTCH3* (FDR = 8.16×10^−9^) were enriched in the *HHEX*-low human T-ALLs but *NOTCH1* was not differentially expressed (FDR = .517) between *HHEX*-high and *HHEX*-low cases (*DTX1* probes are not on the microarray used in this analysis). The *LMO2*-expressing human T-ALLs did not show statistically significant difference in survival but *HHEX*-expressing cases showed significantly worse overall survival compared to nonexpressing cases with current chemotherapy regimens (Log-rank test, *P = .19* for *LMO2* cases and *P = .03* for *HHEX* cases). T-ALL cases that co-expressed both genes showed a trend toward worse overall survival when compared with cases that expressed neither *LMO2* or *HHEX* (*P = .08*, Log rank test).

### *Hhex* expression is recapitulated by endogenous regulatory elements

**Figure 5:**
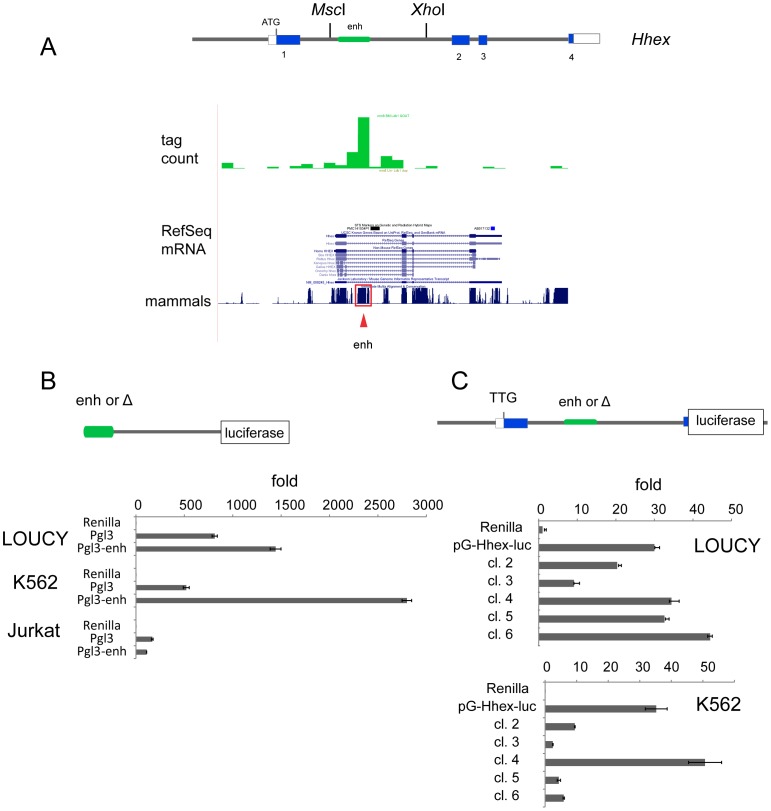
T-ALL expression of *HHEX* requires its promoter and enhancer. (**A**) Schematic shows the structure of the mouse *Hhex* gene with 4 exons (coding sequences shown in blue). We analyzed ChIP-seq data from anti-Ldb1 that showed occupancy within intron 1 (green bar graph). The RefSeq mRNAs for *Hhex* are shown below the sequencing tag counts. The bar graph under this shows the conservation across multiple mammalian species. The red box denotes an enhancer that was previously functionally characterized as specifying blood-specific expression of *Hhex*. (**B**) We cloned the enhancer shown in panel A 5′ to the SV40 promoter and transfected it into LOUCY, K562, and Jurkat cell lines along with pCMV-*Renilla* luciferase vector. Luciferase values were normalized to *Renilla* to correct for transfection efficiency and expressed as fold over *Renilla* alone. (**C**) We constructed a luciferase expression vector with 1 kb of the *Hhex* promoter, exon 1, intron 1, and replaced exon 2 with the luciferase cDNA. We transfected this reporter (pG-*Hhex*-luc) into K562 and LOUCY cells. Clones 1–6 have 50–100 bp deletions 5′ to 3′ within the enhancer region. Luciferase expression was normalized to *Renilla* for transfection efficiency and expressed as fold above that seen in *Renilla* alone.

The genetic evidence from retroviral insertional mutagenesis studies and the *CD2-Lmo2* and human T-ALL gene expression clustering result could be explained if *Hhex* was a direct target of *Lmo2*. Thus, we mined existing anti-Ldb1 ChIP-seq datasets from Lineage^−^ HSPCs for enrichment of sequences near *Hhex* [35]. We found a high number of tags in intron 1 of *Hhex* flanked by *Msc*I and *Xho*I sites (Figure 5A). This region contains an enhancer that drove transgenic reporter expression in blood islands of the embryonic yolk sac in in vivo mouse studies [36]. We cloned this putative enhancer into pGL3, 5′ of the SV40 promoter and transfected it into LOUCY, K562, and Jurkat cells. LOUCY (T-ALL) and K562 (CML) both express endogenous *HHEX* but Jurkat (T-ALL) cells do not. This reporter plasmid showed luciferase expression in LOUCY and K562 cells only (Figure 5B).

We also constructed a reporter with 1 kb of the *Hhex* promoter, exon 1 and intron 1 of *Hhex*, and luciferase substituted for exon 2; the *Hhex* ATG was mutated to TTG. We transfected this reporter (pG-*Hhex-luc*) and observed luciferase expression in K562 and LOUCY, but not in Jurkat cells. We made constructs with 50–100 bp truncations of the enhancer and transfected them into LOUCY and K562 cells. We lost luciferase expression with the first 100 bp deleted (Figure S7 in File S1), but additional truncations and deletion of the entire enhancer (cl.6 in Figure 5C) did not eliminate luciferase expression in LOUCY cells. In contrast, deletion of the enhancer in K562 cells abrogated luciferase expression (cl.6 in Figure 5C). Thus, the *Hhex* enhancer had different roles in LOUCY and K562 cells in transient transfection assays.

### *HHEX* is a transcriptional target of LMO2

**Figure 6:**
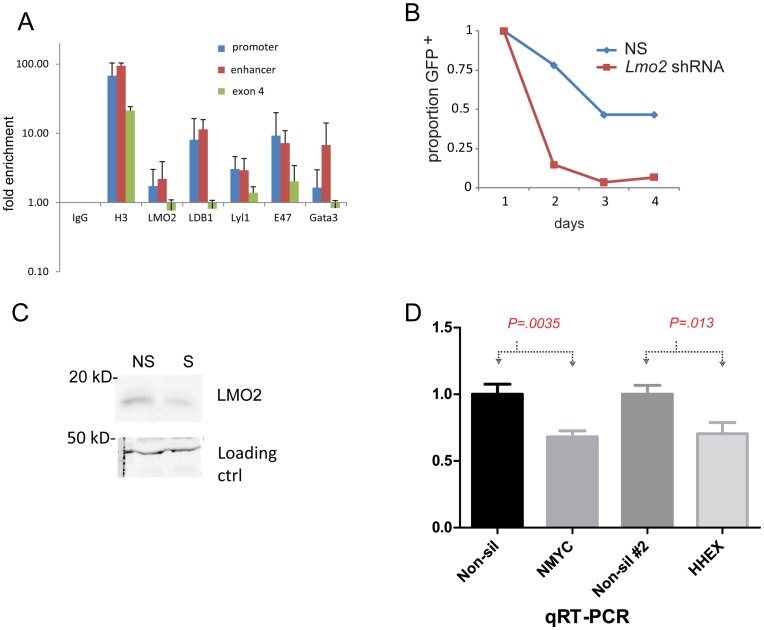
*HHEX* is a direct transcriptional target of an LMO2 protein complex. (**A**) We analyzed the occupancy of LMO2 and its binding partners at the *HHEX* enhancer, promoter, and exon 4 (nonspecific) in LOUCY cells by ChIP. Bar graph shows a summary of 4 independent experiments using antibodies against LMO2, LDB1, LYL1, GATA3, and E47 for ChIP followed by qPCR analysis relative to IgG control. (**B**) Nonsilencing shRNA or specific shRNA directed against *LMO2* was transfected into LOUCY cells followed by sorting for GFP^+^ cells. Cells were counted and analyzed by propidium iodide flow cytometry for viability. Y-axis denotes viable GFP-expressing cells. (**C**) LOUCY cells transfected with nonsilencing (NS) or *LMO2* shRNAs were harvested 24 h after transfection and whole cell lysate prepared. Protein was separated by SDS-PAGE followed by transfer to nitrocellulose and Western blot with monoclonal anti-LMO2. Quantification was performed by infrared-dye conjugated secondary antibodies which showed specific shRNA knocked down LMO2 protein to 51% of the level present in nonsilencing shRNA. (**D**) We harvested mRNA from *LMO2* knockdown LOUCY cells and analyzed *MYCN* and *HHEX* expression by qRT-PCR. Bar graphs show quantity of *MYCN* or *HHEX* expressed relative to LOUCY transfected with non-silencing shRNA. The graph shows a summary of three independent knockdown experiments. The mean values were compared by Mann-Whitney U-test generating the *P* values shown.

The reporter assays suggested to us that both the *Hhex* promoter and enhancer were active in T-ALL but neither sequence contained tandem E boxes or E box-GATA sites as have been described in erythroid promoters[19]. The enhancer had 4 GATA sites and a non-canonical E box (Figure S7 in File S1). We performed chromatin immunoprecipitation (ChIP) using antibodies against LMO2, LDB1, LYL1, GATA3, E47, and H3 in LOUCY cells. We included anti-GATA3 since prior studies had shown that it can bind LMO2 and co-activate target genes [37]. We analyzed by ChIP the *HHEX* promoter, enhancer, or exon 4, which is not predicted to be bound by any of the proteins. Antibodies against LMO2, LDB1, LYL1, and GATA3 showed enrichment for the promoter and enhancer suggesting specific occupancy (Figure 6A). E47 ChIP showed more nonspecific enrichment of exon 4. Next, we knocked down *LMO2* in LOUCY cells with an shRNA lentiviral plasmid that co-expressed GFP. We transfected this construct, sorted GFP^+^ cells and observed their growth *in vitro*. The GFP^+^ cells expressing *LMO2* shRNA did not survive beyond 2 days whereas cells expressing a non-silencing shRNA were maintained over that same time period (Figure 6B). Next, we analyzed LMO2 protein from GFP^+^ LOUCY cells transfected with silencing or nonsilencing shRNA by Western blot. LMO2 protein was decreased by 50% by silencing shRNA (Figure 6C). We performed knockdown transfections with GFP^+^ sorting and isolation of whole RNA from both non-silenced and silenced cells. We analyzed *HHEX* and *MYCN* by qRT-PCR and found that both mRNAs were decreased 40% with *LMO2* knockdown when compared to cells expressing nonsilencing shRNA, a statistically significant result (Figure 6D, Student t test, *P* = .0035 for *MYCN* and *P* = .013 for *HHEX*).

### *Hhex* conditional inactivation prolongs the latency of *Lmo2*-induced T-ALL

**Figure 7:**
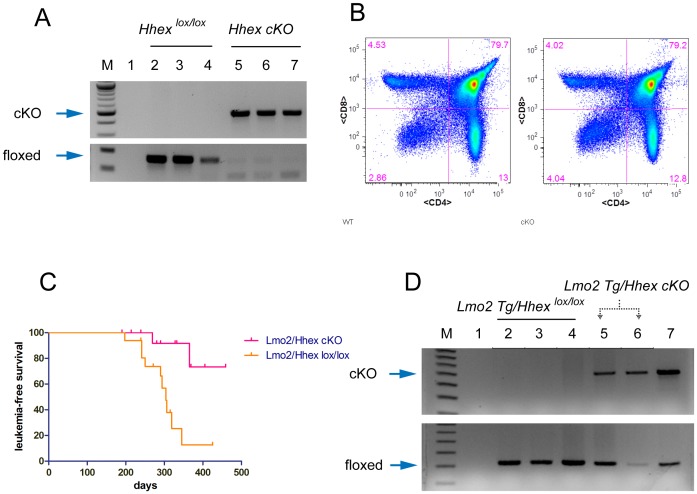
Conditional inactivation of *Hhex* prolongs latency of *Lmo2*-induced T-ALL. (**A**) Genomic DNAs from bone marrow (lanes 2, 5), spleen (3, 6), and thymus (lanes 4, 7) were analyzed using primers specific for floxed *Hhex* or deleted *Hhex* (*cKO*); lane 1 is H_2_O; bands are indicated by blue arrows. Tissues from two representative mice are shown: *Hhex^lox/lox^* and *Hhex^lox/lox^; Vav-iCre* (designated *Hhex cKO*). (**B**) Single cell suspension was prepared from *Hhex^lox/lox^* or *Hhex cKO* thymi and analyzed by flow cytometry for CD4 (x-axis) and CD8 (y-axis) antigen expression. Numbers show the percentage of cells in each quadrant. (**C**) Graph shows survival analysis of *Lmo2/Hhex cKO* and *Lmo2/Hhex^lox/lox^* mice (n = 16 per group). The median survival of *Lmo2/Hhex^lox/lox^* mice was 306 days whereas for *Lmo2/Hhex cKO*, the median was not yet attained. *P* value represents the comparison of survival times by Log-rank test. (**D**) Genomic DNA was prepared from thymi involved with T-ALL of *Lmo2/Hhex^lox/lox^* (n = 3, lanes 2–4) and *Lmo2/Hhex cKO* mice (n = 2, lanes 5, 6); lane 7 shows positive controls for PCR of deleted (cKO) and floxed *Hhex* (blue arrows).

The reporter assays, ChIP experiments, and *LMO2* knockdowns suggested that *HHEX* was a direct transcriptional target of *LMO2*. To analyze the role of *Hhex* in the development of *Lmo2*-induced T-ALL, we bred the *CD2-Lmo2* transgenic mice to floxed *Hhex* (*Hhex^lox/lox^*) or to *Hhex^lox/lox^*; *Vav-iCre* (conditional knockouts termed *Hhex* cKO, Figure S6 in File S1). The *Vav-iCre* transgene which is expressed at E18.5 in all hematopoietic tissues induced highly efficient deletion of *Hhex* in bone marrow, spleen, and thymi and the mice were viable and showed no overt signs of disease (Figure 7A lanes 5-7). The *Hhex* cKO mice had normal leukocyte counts but had major defects in B cells similar to what has been described in chimeric mice made from *Hhex^-/-^* ES cells[38]. Importantly, the thymi of *Hhex* cKOs showed normal cellularity and a normal pattern of T-cell development as analyzed by flow cytometry for CD4 and CD8 antigens (Figure 7B). We observed *CD2-Lmo2* transgenic mice with and without *Hhex* deletion and found a striking difference in T-ALL onset. *CD2-Lmo2* transgenic mice with *Hhex^lox/lox^* died from T-ALL with a median survival of 306 days (Figure 7C). Three out of 16 *CD2-Lmo2* transgenic mice with *Hhex* cKO developed T-ALL. We genotyped the DNA from these T-ALLs and one tumor showed incomplete deletion of the *Hhex* gene (lane 5, Figure 7D) and the others showed complete deletion (lane 6, Figure 7D). *Lmo2*-induced T-ALL onset was significantly prolonged with *Hhex* conditional inactivation (Figure 7C, Log rank test, *P* = .003), suggesting that *Lmo2* induces the expression of *Hhex* to trigger T-ALL.

## Discussion

Transcriptional profiling has provided important insights into the pathophysiology of human T-ALL. In our previous work, we discovered that genes that were concordantly expressed with *LMO2* in human T-ALL were also targets of insertional mutagenesis in murine T-ALL suggesting that they were causal and important in T-ALL induction [26]. We reasoned that gene expression analysis of T-ALLs developing in *CD2-Lmo2* transgenic mice may identify these same cooperating oncogenes. Instead, the *CD2-Lmo2* transgenic T-ALLs were quite different from the AKXD models of *Lmo2*-induced T-ALL. First, we found that *Lyl1* was the predominant bHLH gene upregulated whereas *Tal1* was rarely upregulated. *TAL1* is commonly deregulated in human T-ALL and is co-mutated with *LMO1* and *LMO2*. *TAL1* was mutated in the SCID-X1 gene therapy-induced T-ALLs that had insertional mutation at *LMO2*. In contrast, *LYL1* deregulation was infrequently concordant with *LMO1* and occurred more commonly with *LMO2* [22], [39]. This observation and the findings in our *CD2-Lmo2* transgenic T-ALLs suggest that *LMO2* deregulation in T-cell progenitors may preferentially select for *LYL1* upregulation.

The enforced expression of *Lmo2* by the *CD2* minigene may have an analogous “hit” in human T-cell progenitors deregulating *LMO2* at a specific developmental stage preceding upregulation of *LYL1*. In this study, we also show that LYL1 associates with LMO2 in a macromolecular complex containing LDB1, E47, and other proteins which may autoregulate some of the genes of this complex. In contrast, *TAL1* may be the preferred bHLH gene deregulated if the *LMO2* hit occurs in a hematopoietic stem cell such as in the gene therapy cases or in AKXD mice. The subsequent T-ALLs that develop differ in the cell of origin (immature v. mature) which may explain how *LYL1* and *TAL1* are present in two different T-ALL disease subtypes. *LYL1* is highly expressed in T-ALLs that are derived from the most immature T-cell progenitors which presumably give rise to ETP-ALL occurring more commonly in adults [22], [34], [39]. Lyl1 and Tal1 are similar to each other and both heterodimerize with E2A proteins to bind E boxes. Although there are overlapping targets, there is also evidence for unique targets. For example, *Tal1^-/-^* induces severe defects in primitive hematopoiesis that are not rescued by *Lyl1* [40]. In contrast, there is functional overlap in adult hematopoiesis [41]. More recently, conditional knockouts of *Lyl1* showed major defects in lymphoid-primed multipotent progenitor cells and early T-cell progenitor cells supporting a role for this oncogene in the transformation of these same cell types into ETP-ALL [42].

Oncogenes that are upregulated in ETP-ALLs, *LYL1*, *HHEX*, and *MYCN*, are also upregulated concordantly in a subset of *CD2-Lmo2* transgenic T-ALLs. *MEF2C* is also overexpressed in ETP-ALLs but it was upregulated in only one *CD2-Lmo2* T-ALL, concordant with the only leukemia overexpressing *Tal1*. We previously found that *Mef2c* was a cooperating oncogene with *Lmo2* in AKXD T-ALLs where *Tal1* upregulation was prominent suggesting that *Mef2c* may be co-mutated with *Tal1* in human cases as well [26]. We analyzed the raw ChIP-seq data from the CCRF-CEM cell line done by Sanda et al. CCRF-CEM cells express *TAL1*, *LMO2*, and *HHEX*. The sequencing data showed TAL1 occupancy at the *HHEX* intronic enhancer (Figure S8 in File S1). Interestingly, LMO2 occupancy was observed at the *HHEX* promoter and enhancer similar to our results in LOUCY cells. The concordant expression of *HHEX* and *LMO2* in LOUCY cells is due to direct transcriptional regulation by LMO2 and associated proteins such as LDB1, LYL1 and perhaps GATA3. Our results are similar to data on the MOLT-4 cell lines where a complex of LMO2, FLI1, and ERG binds to the *HHEX* intronic enhancer to regulate its expression; this study also showed that *HHEX* was required by the MOLT-4 cells for their growth and maintenance in culture [43]. Alternatively, human T-ALLs may upregulate *MEF2C* or other transcription factors that have been shown to bind the *HHEX* enhancer in developing hematopoietic progenitor cells [44], [45].

Whatever the mechanism may be for *Hhex* activation, our experiments show that it is an important biomarker in T-ALL and a critical mediator of *Lmo2*'s oncogenic actions. This argument is supported by the remarkable difference in latency of T-ALLs developing in *Lmo2/Hhex* cKO compared with *Lmo2* transgenics and the incomplete deletion of *Hhex* in those T-ALLs that did develop in *Lmo2/Hhex* cKO mice. We also discovered that T-ALL patients with *HHEX* overexpression have a statistically significant worse overall survival. The enforced expression of *Hhex* in bone marrow transduction and transplantation experiments induces highly penetrant T-ALL[46]; and, enforced expression in T-cell progenitors confers increased self-renewal similar to what is observed by enforcing expression of *Lmo2* [29]. *Hhex* encodes a homeobox transcription factor with repressive activities [47], [48]. The conditional knockouts will be highly informative in understanding *Hhex*'s normal and oncogenic functions.

In conclusion, our results suggest that *CD2-Lmo2* transgenic mice develop T-ALL by two distinct pathways. The ETP-ALL-like pathway involves *Lyl1* upregulation and activation of *Hhex* and *Mycn*. The alternate pathway involves upregulation of *Notch1* and its targets which could reflect the developmental stage at which the T-ALLs developed. The *CD2-Lmo2* T-ALLs closely model human T-ALL since human ETP-ALL have decreased incidence of *NOTCH1* mutations and decreased target gene activation. Recently, 12 ETP-ALLs were analyzed by whole genome sequencing and one tumor had an interstitial deletion 5′ of *LMO2* and another had a deletion of *RLIM* which encodes an E3 ubiquitin ligase for the LMO2/LDB1 proteins [20], [49]. These findings directly implicate *LMO2* as a driver in human ETP-ALL. However, most ETP-ALLs have transcriptional upregulation of *LMO2* which could be due to an inability to repress an HSC-like transcriptional program. *LMO2* deregulation in ETP-ALLs may be due to mutations in transcription factors or co-factors that have been recently identified [43], [50]-[52]. This idea is mechanistically similar to the HOXA-mediated activation of *LMO2* in leukemias other than T-ALL [53]. The striking similarities between *CD2-Lmo2* transgenic T-ALLs and human T-ALL suggest that these transgenic mice are a compelling mouse model of human disease, one that will prove useful in studying a highly treatment-resistant leukemia.

## Methods

### Mouse models

The murine *Lmo2* cDNA was cloned into the *CD2* minigene construct, p29ΔZ [54]. The construct was sequence verified and linearized by digestion with *Not*I, purified, and injected into the pronuclei of B6C3HF2 zygotes_ENREF_24. One founder was identified and germline transmission verified by Southern blot analysis of *Eco*RI digested tail DNA and the *Lmo2* cDNA as the probe. Mice were maintained in SPF facilities at NCI-Frederick and at Vanderbilt in accordance with approval from the National Cancer Institute Institutional Animal Care and Use Committee and the Vanderbilt Institutional Animal Care and Use Committee. Initial tumor studies used B6.*CD2-Lmo2* transgenics that were backcrossed N2. More recent studies used B6.*CD2-Lmo2* transgenics that were backcrossed to N11. These mice were crossed with *Hhex^lox/lox^* mice to create *Lmo2* TG/+; *Hhex^lox/lox^*. Floxed Hhex mice were created at NCI Frederick and will be described in detail elsewhere (see supporting information); they were maintained by backcrossing to B6 to N3 and intercrossed to create homozygous floxed mice, *Hhex^lox/lox^*. The *B6.Vav-iCre* transgenics were purchased from Jackson Labs and crossed on to floxed *Hhex* creating two cohorts of mice (i.e. with and without *Vav-iCre*) with the following genotypes and equivalent genetic backgrounds: *Lmo2* TG/+; *Hhex^lox/lox^* and *Lmo2* TG/+; *Hhex^lox/lox^*; *Vav-iCre* TG/+. Morbid mice were sacrificed and hematopoietic tissues harvested and protein, DNA, and RNA prepared as described [12]. *Notch1* mutational analysis is described further in the supporting information file and oligos are listed in Table S2 in File S1.

### Human samples and cell lines

All primary samples were collected by consent and under protocols approved by the Institutional Review Boards of Vanderbilt and St. Jude. This study analyzed Affymetrix gene expression data that was previously published [4], [49]. Kaplan Meier analysis was applied to the overall survival data on T-ALL patients enrolled in St. Jude's Total Therapy program for childhood acute lymphoblastic leukemia. Seventy eight patients were analyzed for gene expression analysis of whom 6 were on study 13B and the rest on study 15 [55], [56]. Other primary samples were provided de-identified by the Children's Oncology Group (COG) and by the tumor bank at Vanderbilt Ingram Cancer Center. All studies were reviewed by the Vanderbilt Institutional Review Board which deemed the work non-human subject research; the Vanderbilt IRB waived the need for consent. LOUCY and Jurkat E6.1 were purchased from ATCC and maintained in RPMI 1640 with 10% fetal calf serum and 1% penicillin/streptomycin in 5% CO_2_ at 37°C. Gel shift assay, chromatin immunoprecipitation, and reporter analysis are described in detail in file S1.

### Gene expression analysis

Human T-ALL RNA was analyzed by labeling, hybridization, and analysis off of the Affymetrix U133A chip as previously described and deposited in GEO (GDS4299) [4]. Quantitative PCR throughout this study was done on tumor cDNA using custom Taqman probes (Invitrogen) and normalized to whole thymus RNA (see Table S1in File S1 for probe list).

### Nuclear extracts and gel shift assay

Our extraction protocol is modified from the original Dignam et al method [57]. Cells (5×10^7^) were washed twice with 10 ml of cold PBS, resuspended in 5 packed cell volumes of buffer A (10 mM HEPES, 1.5 mM MgCl2, 10 mM KCl, 50 mM sucrose, 0.1% NP-40, 0.5 mM DTT, 0.5 mM PMSF, pH 7.9) and incubated on ice for 10 min. The nuclei were pelleted by centrifugation at 1000 *g* for 5 min, and resuspended in 2 packed nuclear volumes of buffer C (20 mM HEPES, 1.5 mM MgCl_2_, 420 mM KCl, 0.2 mM EDTA, 25% v/v glycerol, 0.5 mM DTT, 0.5 mM PMSF, pH 7.9). The resulting nuclear suspension was rocked at 4°C for 2 h. The sample was centrifuged at 13 000 *g* for 10 min, and aliquots of the nuclear extract were frozen immediately on dry ice. Samples were stored at −80°C. The protein concentration of the nuclear extract was determined by the Bradford assay (BioRad). EMSAs were performed with ^32^P-labelled double-stranded oligonucleotides as described [58]. The sequence of the sense strands of individual oligonucleotides is as follows: E-Ebox, AGCCGACCATCTGTTCAGGTAAACAGATGGTCGACA and E-EΔ, AGCCGACCATCTGTTCAGGTAAACCGATCGTCGACA. LOUCY nuclear extract (5 mg) was incubated for 20 min at 25°C in binding buffer (25 mM HEPES, 100 mM KCl, 0.6 mM EDTA, 10% v/v glycerol, 2.8 mM DTT, pH 7.9) in the presence of 3 mg/ml poly(dI-dC) and 20 nM ^32^P-labelled oligonucleotide with or without antibodies: anti-LMO2 mouse monoclonal (kindly provided by Dr. Ron Levy, Stanford); anti-LDB1 from Santa Cruz (sc-11198), anti-LYL1 from Santa Cruz (sc-46158), anti-E47 from Santa Cruz (sc-763). Samples were loaded onto 6% native polyacrylamide gels, which were run for 12 h at 11 mA constant current in TGE running buffer (50 mM Tris HCl, 380 mM glycine, 2 mM EDTA). After drying the gel, the DNA–protein complexes were visualized by autoradiography.

### Chromatin immunoprecipitation analysis

LOUCY cells (5×10^6^ per antisera used) were washed once in PBS and cross linked with 2% formaldehyde for 5 min at 37°C followed by quenching with 10% glycine for 10 minutes at 37°C. Lysate was prepared by suspending the cells in lysis buffer (50 mM HEPES (pH 7.8), 140 mM NaCl, 1 mM EDTA, 1% Triton X-100, 0.1% sodium deoxycholate, and a mixture of protease inhibitors (Roche). Lysate was sonicated with 2×5-sec pulses at 4 watts with a 20 s refractory period in between the sonications, to reduce the chromatin fragments to ∼500 bp. Clarified lysates were pre-cleared with Protein A-agarose (Santa Cruz Biotechnology Inc.) for 30 min and immunoprecipitated with 5 µg of the indicated antisera (same as for EMSA). Following incubation with protein A-agarose (30 min), immune complexes were washed once with low salt (20 mM Tris HCl, 150 mM NaCl, 1% Triton X-100, 2 mM EDTA, pH 8.1), high salt (20 mM Tris HCl, 500 mM NaCl, 1% Triton X-100, 2 mM EDTA, pH 8.1), and LiCl immune complex wash buffer (10 mM Tris HCl, 250 mM LiCl, 0.5% NP-40, 0.5% sodium deoxycholate, pH 8.1), and eluted with 400 µl elution buffer (1% SDS, 0.1 M NaHCO_3_). Cross links were reversed with 5 M NaCl at 60°C, DNA was recovered by ethanol precipitation, the mixture treated with proteinase K digestion, and the DNA was purified using a PCR purification kit (Qiagen), and analyzed by real time PCR using the primers listed in Table S4 in File S1 (BioRad).

## Supporting Information

File S1 **Supporting Information. Table S1.** Taqman probes used for qRT-PCR. **Table S2.** Primers used for Notch1 mutational analysis. **Table S3.** Summary of Notch1 mutational analysis. **Table S4.** List of primers used in ChIP assay. **Figure S1.** Supervised clustering of human T-ALL based on LMO2. **Figure S2.** Supervised clustering of human T-ALL based on HHEX. **Figure S3.** Concordant expression of HHEX, LMO2, and MEF2C in primary T-ALLs. **Figure S4.** Survival analysis differs among CD2-Lmo2 transcriptional profiles. **Figure S5.** Crossing CD2-Lmo2 transgenics onto B6 increases T-ALL latency. **Figure S6.** Hhex conditional knockout alleles. **Figure S7.** Enhancer constructs analyzed in paper. **Figure S8.** ChIP-seq data from CCRF-CEM cells.
(PDF)
