# Supplementary material for: LIM Domain Only-2 (LMO2) Induces T-Cell Leukemia by Two Distinct Pathways
Source: PLoS One. 2014 Jan 21;9(1):e85883. doi: 10.1371/journal.pone.0085883 (PMC3897537; doi:10.1371/journal.pone.0085883)
Supplement: File S1 — Supporting Information. Table S1. Taqman probes used for qRT-PCR. Table S2. Primers used for Notch1 mutational analysis. Table S3. Summary of Notch1 mutational analysis. Table S4. List of primers used in ChIP assay. Figure S1. Supervised clustering of human T-ALL based on LMO2. Figure S2. Supervised clustering of human T-ALL based on HHEX. Figure S3. Concordant expression of HHEX, LMO2, and MEF2C in primary T-ALLs. Figure S4. Survival analysis differs among CD2-Lmo2 transcriptional profiles. Figure S5. Crossing CD2-Lmo2 transgenics onto B6 increases T-ALL latency. Figure S6. Hhex conditional knockout alleles. Figure S7. Enhancer constructs analyzed in paper. Figure S8. ChIP-seq data from CCRF-CEM cells. (PDF) [file pone.0085883.s001.pdf]

SUPPLEMENTAL METHODS AND FIGURES

Patient samples, qRT-PCR, and gene expression analysis

Primary human T-ALL samples were provided de-identified by the Childrens Oncology Group (COG), and from tissue banks at Vanderbilt and St. Jude. Cells were stored in liquid nitrogen and rapidly thawed at 37°C and transferred to pre-warmed complete media (RPMI 1640, 10% FBS, 1% Penn/Strap). Two hours later, we added 1 ml Tri-Reagent and stored at -80°C for RNA, gDNA & Protein isolation. RNA, DNA & Protein were isolated from 23 human patient T-ALLs and 4 T-ALL cell lines Tall104, Loucy, Jurkat, JM using Ambion TRI-Reagent Cat # AM97JB as per company protocol. The RNA samples were quantified in 10 mM Tris-HCl on the Nanodrop ND-1000. Samples were then analyzed for quality using an Agilent 2100 Bioanalyzer. Samples set up for reverse transcription in duplicate with random hexamer primer and Invitrogen's Superscript II. Reactions were pooled for clean up using Qiagen PCR purification kit (#28104). Real Time PCR reactions were done in triplicates. Microarray data were normalized using the Robust MultiChip Averaging (RMA) algorithm[1] as implemented in the Bioconductor package *Affy*. For pairwise group comparisons, we used t-test in the *Limma* package[2] in Bioconductor to identify differentially expressed probe sets between the *LMO2*-high expression and -low expression groups under comparison. The implementation of t-test in *Limma* uses an empirical Bayes method to moderate the standard errors of the estimated log-fold changes. A false discovery rate of 0.01 was used as a cut-off to identify 130 significant probes for *LMO2* and 122 probes for *HHEX* comparisons. The hierarchical clustering with complete linkage and Euclidean distance was applied to generate the heatmaps shown for identified probes.

Notch1 mutational analysis

We used heteroduplex analysis as previously described [3] and direct Sanger sequencing to identify mutations in exons 26, 27, and 34 of the murine *Notch1* gene. Genomic DNA was extracted from T-

ALLs by homogenization and the Promega SV Genomic Purification Kit (Cat # A2360). Notch1 mouse Exons 26, 27, and 34 were PCR amplified using primer pairs in Table S2. Direct Sanger Sequencing was performed on PCR products by the Vanderbilt Core Sequencing Facility. Mutations were analyzed using Invitrogen Vector NTI Version 11.0 and novoSNP, using ABI trace files as input [4]. The analysis was conducted using the lowest possible SNP detection threshold (Score =1, FScore = 1), which is intended to minimize false negatives. Additionally, all traces, as aligned by novoSNP, were analyzed manually.

Nuclear Extracts and gel shift assay

Our extraction protocol is modified from the original Dignam et al method [5]. Cells ($5X10^7$) were washed twice with 10 ml of cold PBS, resuspended in 5 packed cell volumes of buffer A (10 mM HEPES, 1.5 mM MgCl2, 10 mM KCl, 50 mM sucrose, 0.1% NP-40, 0.5 mM DTT, 0.5 mM PMSF, pH 7.9) and incubated on ice for 10 min. The nuclei were pelleted by centrifugation at 1000 *g* for 5 min, and resuspended in 2 packed nuclear volumes of buffer C (20 mM HEPES, 1.5 mM $MgCl_2$, 420 mM KCl, 0.2 mM EDTA, 25% v/v glycerol, 0.5 mM DTT, 0.5 mM PMSF, pH 7.9). The resulting nuclear suspension was rocked at 4° C for 2 h. The sample was centrifuged at 13 000 *g* for 10 min, and aliquots of the nuclear extract were frozen immediately on dry ice. Samples were stored at -80° C. The protein concentration of the nuclear extract was determined by the Bradford assay (BioRad). EMSAs were performed with ^{32}P-labelled double-stranded oligonucleotides as described [6]. The sequence of the sense strands of individual oligonucleotides is as follows:

E-Ebox, AGCCGACCATCTGTTCAGGTAAACAGATGGTCGACA

E-EΔ, AGCCGACCATCTGTTCAGGTAAACCGATCGTCGACA

LOUCY nuclear extract (5 mg) was incubated for 20 min at 25° C in binding buffer (25 mM HEPES, 100 mM KCl, 0.6 mM EDTA, 10% v/v glycerol, 2.8 mM DTT, pH 7.9) in the presence of 3 mg/ml poly(dI-dC) and 20 nM ^{32}P-labelled oligonucleotide with or without antibodies: anti-LMO2 mouse monoclonal (kindly provided by Dr. Ron Levy, Stanford); anti-LDB1 from Santa Cruz (sc-11198), anti-LYL1 from Santa Cruz (sc-46158), anti-E47 from Santa Cruz (sc-763). Samples were loaded onto 6% native polyacrylamide gels, which were run for 12 h at 11 mA constant current in TGE running buffer (50 mM Tris HCl, 380 mM glycine, 2 mM EDTA). After drying the gel, the DNA–protein complexes were visualized by autoradiography.

Chromatin Immunoprecipitation analysis

LOUCY cells (5×10^6 per antisera used) were washed once in PBS and cross linked with 2% formaldehyde for 5 min at 37°C followed by quenching with 10% glycine for 10 minutes at 37°C. Lysate was prepared by suspending the cells in lysis buffer (50 mM HEPES (pH 7.8), 140 mM NaCl, 1 mM EDTA, 1% Triton X-100, 0.1% sodium deoxycholate, and a mixture of protease inhibitors (Roche). Lysate was sonicated with 2x5-sec pulses at 4 watts with a 20 s refractory period in between the sonications, to reduce the chromatin fragments to ~ 500bp. Clarified lysates were pre-cleared with Protein A-agarose (Santa Cruz Biotechnology Inc.) for 30 min and immunoprecipitated with 5 µg of the indicated antisera (same as for EMSA). Following incubation with protein A-agarose (30 min), immune complexes were washed once with low salt (20 mM Tris HCl, 150 mM NaCl, 1% Triton X-100, 2 mM EDTA, pH 8.1), high salt (20 mM Tris HCl, 500 mM NaCl, 1% Triton X-100, 2 mM EDTA, pH 8.1), and LiCl immune complex wash buffer (10 mM Tris HCl, 250 mM LiCl, 0.5% NP-40, 0.5% sodium deoxycholate, pH 8.1), and eluted with 400 µl elution buffer (1% SDS, 0.1M $NaHCO_3$). Cross links were reversed with 5M NaCl at 60°C, DNA was recovered by ethanol precipitation, the mixture treated with

proteinase K digestion, and the DNA was purified using a PCR purification kit (Qiagen), and analyzed by real time PCR (BioRad).

Cell culture and knockdown

The human K562 (CCL-243)**,** Jurkats, Tall-104 (CRL-11386) & Loucy (CRL-2629) cells were obtained from the American Type Culture Collection. The Loucy and Jurkat cells were grown in RPMI-1640 medium and K562 were grown in IMDM containing 10% fetal bovine serum, 1% Penn/Strep, in a humidified atmosphere of 5% CO_2 in air at 37°C, in T25 or T75 flasks. Knockdown of LMO2 was done by electroporation of a pGIPZ construct expressing a specific shRNA (i.e. LMO2, cat# V2LHS_62634) or non-silencing control into LOUCY cells using manufacturer's conditions (Lonza). We electroporated $2x10^6$ Loucy cells with 5 µg of pGIPZ DNA in solution V, kit VCA-1003. We added 50 µL of complete growth media to the cuvettes and transferred the cells to a 12 well plate with 1ml prewarmed media per well. Cells were then sorted for GFP positivity and counted for viability or whole lysate prepared using RIPA buffer (2mM EDTA, 150mM NaCl, 1% NP-40, 0.5% Sodium Deoxycholate,0.1% SDS, 50mM HEPES buffer pH 7.4, 1 mM DTT, 1 tablet of Roche complete mini protease inhibitor ref # 05892970001) for Western blot.

Luciferase reporter constructs

First, we PCR amplified a 4 kb fragment using mouse spleen cDNA as template and Expand Long Template kit (Roche Applied Science). Forward primer sequence included a *Kpn*I site 5' of 1 kb of the Hhex promoter: 5'- GGTACCTTATTCTGAAAGATGTCCGTGACTCC-3'. Reverse primer included part of exon 2 followed by *Hind*III site: 5'- CGAAGCTTGGTATTTCTGAGTCTCGAACTTCTTCTCCAGCTC-3'. This 4 kb fragment was TA-cloned into pGEMTEasy and completely sequence verified and designated pGEMTEasy-Hhex. We used this plasmid to create pHhex-enh-SV40 and pG-Hhex-luc. For pHhex-enh-SV40, we cut out a 1.2 kb fragment of Hhex intron 1 that included the 409 bp enhancer with

*Mlu*I and *Xho*I and cloned it into pGL3 double digested with same enzymes.To construct pG-Hhex-luc, we removed the luciferase cDNA and SV40 poly(A) signal from pGL3-basic using *Xho*I/*Sal*I and cloned it into the *Sal*I digested pGEMTEasy-Hhex construct noted above. Thus, the luciferase cDNA was positioned immediately 3' of a partial Hhex exon 2. This was designated pG-Hhex-luc which served as template for enhancer deletions.

Luciferase reporter assays

Jurkat and K562 cells were transfected using Lipofectamine 2000 transfection reagent (Life Technologies, cat # 11668-019) using manufacturer's protocols; in a 6-well plate, we transfected 300 ng of the reporter DNA pG-*Hhex*-luc per dish in a 6 well plate in triplicate along with pCMV-*Renilla* 25 ng and 5 ng for Jurkats & K562 respectively. For Loucy cells, we electroporated $2x10^6$ cells in triplicate using Lonza nucleofector technology with solution V, kit VCA-1003. We transfected 300 ng of pG-*Hhex*-luc & 25 ng pCMV-*Renilla* and immediately added 50 µL of complete growth medium to the cuvettes and transferred the cells to a 12 well plate with pre-warmed media. After 48 h, cells were washed, lysed in passive lysis buffer (Promega), and activity determined using Dual-Luciferase Reporter Assay System (cat # E1910) according to the manufacturer's instructions.

Fluorescent in situ hybridization

LMO2 BAC (2533C16) and MEF2C BAC (RP11690G22) were purified using Qiagen maxiprep Kit (Cat # 12163), as per the manufacturer's protocol. We labeled 1 ug of each BAC with Spectrum Green dUTP or with Spectrum Orange dUTP using the Vysis Nick Translation Kit (Cat # 32-801300). Cytospins of primary human T-ALL cells from case 05-101 were prepared at 800rpm for 5 min and fixed in 3:1 glacial acetic acid and methanol for 20 min, dehydrated stepwise in 70%, 85% and 100% ethanol for 2 min each, then air dried. For probe precipitation, we added 5 ul of each of the labeled BACs, 10 ul of human Cot-1 DNA (Invitrogen Cat # 15279-101), $1/10^{th}$ volume of 3M Sodium acetate and 2.5x

volume of 100% Ethanol and incubated at -80°C for 30 min. The pellet was spun at 15K for 20 min at 4 °C; supernatant discarded. DNA pellet was washed with 500ul 70% EtOH and resuspended in 10 µl Hybridization buffer (Vysis Cat # 32-131018), then, incubated 1 h in dark at 37°C. We applied 10 µl of labeled probe to each slide including control metaphase slide in the Vysis Hybrite for 73°C 6min, then at 37°C overnight. Slides were washed in SSC and detergent, air dried, and treated with10 µl of DAPII (Vysis Cat #32-804831) counter stain.

Hhex conditional knockouts

The floxed *Hhex* mice will be described in detail elsewhere. Briefly, the murine *Hhex* gene was cloned from a 129 BAC by retrieval methods previously described[7]. A single *loxP* and a *loxP* plus *Frt*-flanked neo cassette were targeted to the resulting construct in two steps in EL350 cells through recombineering. First, to insert the single 5′ *loxP* site, a targeting cassette containing *Pgk-em7-neo* flanked by homology arms to 1st intron of *Hhex* was constructed in PL400. The homology arms are PCR amplified using the following primers: 5′-arm sense, 5′- CGC GAA GCT TGC AGA ACA TGA GTG TGA CCG-3′; 5′-arm antisense, 5′- CGC GGA ATT CAT GAG AGC ACT TCC CAA GGC-3′; 3′-arm sense, 5′- CGC GCT CGA GAA AGA GCG CAC CCT GAG TCT -3′; 3′-arm antisense, 5′- CGC GGG ATC CCC AGA ACG CAA CCA TGT TCC -3′. The homology arms were sequence verified, restriction digested, and cloned into PL400 via four-way ligation. The targeting cassette was released by *BamH*I/*Hind*III double digest and targeted through co-electroporation into heat shock-induced EL350 cells. The *Pgk-em7-neo* sequence was then removed by electroporation into arabinose-induced Cre-expressing EL350 cells, leaving behind a single *loxP* site. To insert the second *loxP* site after exon 4 of *Hhex*, a targeting cassette containing *frt-Pgk-Em7-neo-frt-loxP* flanked by homology arms to targeting site was constructed in PL451. Homology arms were amplified using the following primers: 5′-arm sense, 5′- CGC GAA GCT TCC TAA ACA TGA CAC CTA AAG -3′; 5′-arm antisense, 5′- CGC GGA

ATT CCA CCC TGC TTG GTC CTC TTC-3′; 3′-arm sense, 5′- CGC GGG ATC CGA TTG GAG CTG CCA CTG AGT -3′; 3′-arm antisense, 5′- CGC GGC GGC CGC AGC AGC TGG AAC CTG ACA AC -3′. The targeting cassette was released by *Not*I/*Hin*dIII double digest and targeted similarly as described above. The conditional targeting vector was then linearized by *Not*I digestion and electroporated into 129-derived CJ7 embryonic stem (ES) cells, using standard procedures. G418 (180 µg/ml) and ganciclovir (2 µm) double-resistant clones were analyzed by Southern blotting hybridization, using both 5′ and 3′ external probes. External probes were PCR amplified using the following primers: 5′ probe, sense, 5′- CCC CAC TAC ACC TGG CTA AC -3′; 5′ probe, antisense, 5′- ACG TGG ATG GTA TCA AAG CC -3′; 3′-probe, sense, 5′- GGG ATT TGT TGT TGC TGT GC-3′; 3′-probe, antisense, 5′- CTG GAT GCT GGT GAC TCA GA -3′. Correctly targeted clones were then injected into C57BL/6 blastocysts using standard procedures, and resulting chimeras were mated with C57BL/6 females to obtain germline transmission of the targeted allele. The Neo cassette was removed by crossing to the *Flp* recombinase strain.

Table S1. Taqman probes used for qRT-PCR.

Table S2. Primers used for *Notch1* mutational analysis. Notch1 exons 26, 27, and 34 were amplified using the primers shown.

Table S3. Summary of *Notch1* mutational analysis. *CD2-Lmo2* transgenic T-ALLs were analyzed for *Notch1* mutations by heteroduplex analysis and direct Sanger sequencing. Mutations were found in all the tumors by these methods, most commonly insertions and deletions in exon 34 that were predicted to disrupt the PEST degradation signaling[8].

Table S4. List of primers used in ChIP assay.

Figure S1. Supervised clustering of human T-ALL based on *LMO2*. The Limma package was used to perform clustering analysis based on LMO2 expression, high versus low, with the cutoff being the median expression value. The boxed areas show the probes for LYL1 and HHEX. The black arrows denote cases of ETP-ALL. Color denotes the raw z score for expression.

Figure S2. Supervised clustering of human T-ALL based on *HHEX*. The Limma package was used to perform clustering analysis based on *HHEX* expression, high versus low, with the cutoff being the median expression value. Color denotes the raw z score for expression.

Figure S3. Concordant expression of *HHEX*, *LMO2*, and *MEF2C* in primary T-ALLs. (A) Bar graph shows relative expression of *HHEX* (blue), *MEF2C* (red), and *LMO2* (green) in 23 primary T-ALL samples and 3 cell lines by qRT-PCR using Taqman probes. We analyzed the primary T-ALL samples

by fluorescent *in situ* hybridization (FISH) using labeled BACs but did not find amplification of *HHEX* or *MEF2C* that may account for overexpression. However, 05-101, a case of T-ALL with normal cytogenetics showed evidence for *LMO2* duplication. This T-ALL case had the highest levels of *HHEX*, *MEF2C*, and *LMO2* mRNA by qRT-PCR. **(B)** top panel shows a normal lymphocyte probed with a labeled BAC for *LMO2* and *MEF2C* (spectrum orange); bottom panel shows an interphase FISH of T-ALL 05-101 which had very high levels of all three genes. Red (control) probe showed two signals in all cells whereas *LMO2* probe showed multiple green cells in all leukemic cells consistent with duplication of the gene.

Figure S4. Survival analysis differs among *CD2-Lmo2* transcriptional profiles. We divided the mice developing T-ALL based on their median values of Hhex or Dtx1 mRNA by qRT-PCR. **(A)** shows survival analysis between two cohorts of mice, those with *Hhex* expression (dark gray, n=11) and those without *Hhex* expression (light gray, n=12). **(B)** shows survival analysis based on the expression of *Dtx1*-high (n=12) versus *Dtx1*-low (n=7) . Log-rank analysis was used to generate the *P* values shown.

Figure S5. Crossing *CD2-Lmo2* transgenics onto B6 increases T-ALL latency. All mice from the cohorts shown in Figure 7 were backcrossed to C57BL6 for more than 10 generations which increased T-ALL latency but did not affect penetrance. *CD2-Lmo2* transgenic mice were grouped according to the number of backcrosses to C57Bl6 mice. Thirteen mice at N8-N11 were compared to 32 mice at N2-N4 for onset of T-ALL. Survival analysis is shown and the curves were compared by Log-rank analysis which showed a statistically significant difference in survival, *P*=.04. Median survival for N8-N11 was 255 days and 213 days for N2-N4.

Figure S6. *Hhex* conditional knockout alleles. Schematic shows the wild type *Hhex* gene with 4 exons shown in blue. The description of the targeting vector is provided above and the resulting

targeted allele in ES cells is shown. The ES cells were used to make chimeric mice which were then bred to *Flp* recombinase mice to delete the neomycin cassette resulting in the conditional floxed allele. These mice were backcrossed to B6 and intercrossed to create *lox/lox* homozygous mice. These were bred to *Vav-iCre* transgenic mice to effect conditional inactivation of *Hhex*.

Figure S7. Enhancer constructs analyzed in paper. The intronic 400 bp enhancer in intron 1 of mouse Hhex is shown. The deletion constructs, clones (cl.1-6) transfected in our paper were 50-100 bp deletions from the 5' end of the enhancer as shown. Motifs for specific transcription factor binding sites are shaded and GATA sites are numbered.

Figure S8. ChIP-seq data from CCRF-CEM cells. We mapped the sequence data from anti-LMO2 and anti-TAL1 ChIPs published by Look and colleagues from the T-ALL cell line. These cells express HHEX. In addition to LMO2 and 2 TAL1 ChIP-seq tracks, whole cell extract (WCE), and GATA3 ChIP-seq datasets were also mapped to the human genome (hg18). The black peaks correspond to enriched sequence tags. The red box shows the *HHEX* intron 1 enhancer. The data suggest specific co-occupancy of LMO2 and TAL1 at the *HHEX* enhancer. LMO2 shows occupancy at the *HHEX* promoter as well.

Table S1. Taqman probes used for qRT-PCR.

Assay ID	Target mRNA	Refseq
Mm00456428_m1	*Adam17,mCG8913*	NM_009615.5
Mm00494449_m1	*Cdkn2a,mCG123699*	NM_001040654.1|NM_009877.2
Mm00492297_m1	*Dtx1,mCG131634*	NM_008052.2
Mm00468977_m1	*Ets2,mCG2219*	NM_011809.2
Mm00484682_g1	*Gata1,mCG3964*	NM_008089.1
Mm00492302_g1	*Gata2,mCG130615*	NM_008090.3
Mm01337569_m1	*Gata3,mCG5026*	NM_008091.2
Mm01342805_m1	*Hes1,mCG126604*	NM_008235.2
Mm00439311_g1	*Hes5,mCG3930*	NM_010419.2
Mm00516558_m1	*Heyl,mCG5537*	NM_013905.2
Mm00433954_m1	*Hhex*	NM_008245.3
Mm01340214_m1	*Il2ra,mCG9569*	NM_008367.2
Mm01281681_m1	*Lmo2,mCG9639*	NM_008505.3
Mm00493219_m1	*Lyl1,mCG5919*	NM_008535.1
Mm00600423_m1	*Mef2c,mCG130733*	NM_025282.1
Mm00487803_m1	*Myc,mCG1625*	NM_010849.4
Mm00476449_m1	*Mycn,mCG129638*	NM_008709.2
Mm00435248_m1	*Notch1,mCG11364*	NM_008714.2
Mm01210556_m1	*Olig2,mCG11445*	NM_016967.2
Mm02525085_s1	*Olig3,mCG54581*	NM_053008.2
Mm00478361_m1	*Ptcra,mCG2682*	NM_011195.1
Mm00441144_g1	*Rorc,mCG5011*	NM_011281.1
Mm01270606_m1	*Sfpi1,mCG13483*	NM_011355.1
Mm01187033_m1	*Tal1,mCG16164*	NM_011527.1
Mm00447439_s1	*Tal2,mCG2742*	NM_009317.2
Mm00441699_m1	*Tcf12,mCG16396*	NM_011544.1
Mm01175588_m1	*Tcfe2a,mCG145500*	NM_011548.3
**Human probes**		
Hs00277106_m1	*LMO2,hCG26502*	NM_005574.2
Hs01554603_m1	*MEF2C,hCG36839*	NM_002397.2
Hs01074519_m1	*HHEX,hCG1781169*	NM_002729.2

Table S2. Primer pairs used to amplify *Notch1* exons for mutational analysis. M13 tags (uppercase) were introduced to facilitate sequencing.

Target	Forward	Reverse
Exon 26	GTTTTCCCAGTCACGACaattctatccctgcccatt	CAGGAAACAGCTATGACggtgaatactacctcccctga
Exon 27	GTTTTCCCAGTCACGACtaaggaaaaggggtgctgtg	CAGGAAACAGCTATGACtgcagaggtcagaaagtgttg
Exon 34	CCCCCAGGCCATCCATCATCAA	CAGGAAACAGCTATGACCCGGCTACCTCCAGCCAGTGC
Exon 34	GTT TTCCCAGTCACGACGCTTGTGGTAGCAAGGAAGC	CAGGAAACAGCTATGACACTGAGGTGTGGCTGTGATG
Exon 34	TGTAAAACGACGGCCAGTATAGCATGATGGGGCCACTA	CAGGAAACAGCTATGACACTGGAAAGGACTCCCAACA

Table S3. Summary of *Notch1* mutational analysis. We found evidence for mutations in all the T-ALLs analyzed. The cloned or characterized mutations are shown in the table below. Blank cells indicate that the mutations were found by heteroduplex analysis but were not further characterized by sequencing.

tumor name	Hhex expression	Notch1 mutation	exon 26	exon 27	exon 34
31001	+	+			uncharacterized mutation by duplex
30870	+	+	wt	wt	c.7624_7625insCC; fs stop at T2458
03007		+			wt
30840	+	+	wt	wt	c7347_7348insCT; fs stop at 2397
30831	+	+			
30823	+	+	wt	wt	c.7420_7421insG; fs stop at 2491
30839	+	+			uncharacterized mutation by duplex
30844	+	+	wt	wt	c.7348_7349insAGGGG; fs stop at 2398
31003	+	+			
30847	+	+			uncharacterized mutation by duplex
30991		+	wt	wt	c.7347_7348insC; fs stop at 2491
30832	+	+			uncharacterized mutation by duplex
30843		+	wt	c.G5052GGG, pQ1684insG	c.7347_7348insCCCT; fs stop at 2492
30818		+	wt	c.T5009A, p.I1670N	c.7347_7348insC; fs stop at 2491
30819		+			
30982		+	wt	wt (duplex)	
31388		+	wt	wt	c.7317_7318insG; fs stop at 2491
30830		+	wt	c.T5009A, p.I1670N	c.7347_7348insT; fs stop at 2491
31416		+		wt	wt by duplex
30846		+		wt	wt by duplex
32010		+	wt	wt	c.7624_7625insCC; fs stop at 2458
31647		+			uncharacterized mutation by duplex
03020		+			c.7458_7459insA; fs stop at 2398
31415		+	wt	wt (duplex)	wt
32080		+			c.7459_7480ins 64 bp duplication
03027		+			c.T7085A:p.L2362Q,

Table S4. Primers used for ChIP assay.

Name	sequence
HHEX promoter, forward	AGAATCCAGTTAATTGGGATGATATGAGGC
HHEX promoter, reverse	CCACTTTGAATATAACAATTCGTGAGTCCA
HHEX enhancer, forward	TACGGGGCTGGACCTGGTTCAA
HHEX enhancer, reverse	CGGCTATCAGAAGTCGAGTGTTTCCT
HHEX exon 4, forward	AGTACCTGTTTGACCAAGGTGTTAAGGGGA
HHEX exon 4, reverse	CACGGACGGCTTTATTTTATAGTCAAGTTA

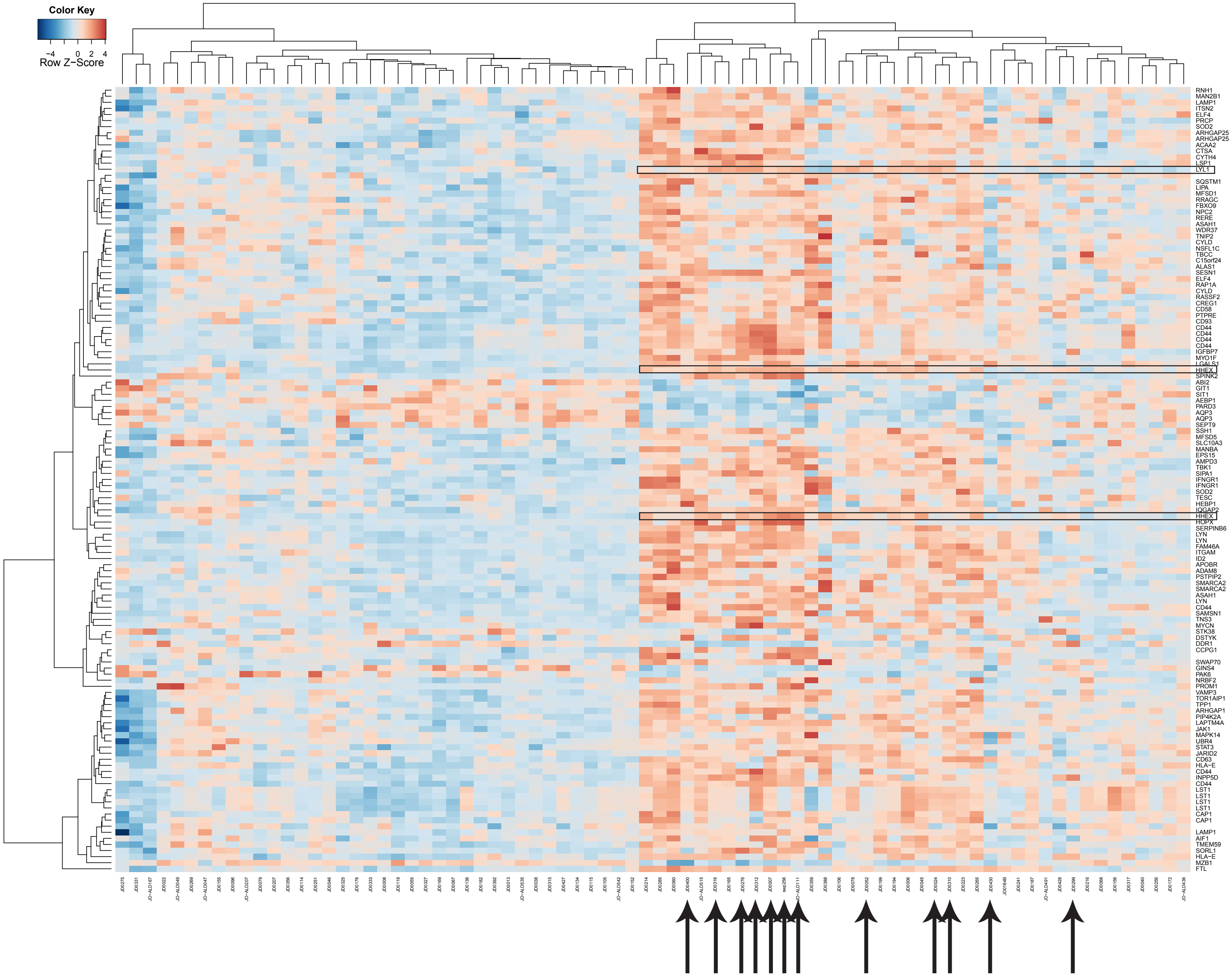
Color Key
−4 0 2 4
Row Z−Score
RNH1
MAN2B1
LAMP1
ITSN2
ELF4
PRCP
SOD2
ARHGAP25
ARHGAP25
ACAA2
CTSA
CYTH4
LSP1
LYL1
SQSTM1
LIPA
MFSD1
RRAGC
FBXO9
NPC2
RERE
ASAH1
WDR37
TNIP2
CYLD
NSFL1C
TBCC
C15orf24
ALAS1
SESN1
ELF4
RAP1A
CYLD
RASSF2
CREG1
CD58
PTPRE
CD93
CD44
CD44
CD44
CD44
IGFBP7
MYO1F
LGALS1
HHEX
SPINK2
ABI2
GIT1
SIT1
AEBP1
PARD3
AQP3
AQP3
SEPT9
SSH1
MFSD5
SLC10A3
MANBA
EPS15
AMPD3
TBK1
SIPA1
IFNGR1
IFNGR1
SOD2
TESC
HEBP1
IQGAP2
HHEX
HOPX
SERPINB6
LYN
LYN
FAM46A
ITGAM
ID2
APOBR
ADAM8
PSTPIP2
SMARCA2
SMARCA2
ASAH1
LYN
CD44
SAMSN1
TNS3
MYCN
STK38
DSTYK
DDR1
CCPG1
SWAP70
GINS4
PAK6
NRBF2
PROM1
VAMP3
TOR1AIP1
TPP1
ARHGAP1
PIP4K2A
LAPTM4A
JAK1
MAPK14
UBR4
STAT3
JARID2
CD63
HLA−E
CD44
INPP5D
CD44
LST1
LST1
LST1
LST1
CAP1
CAP1
LAMP1
AIF1
TMEM59
SORL1
HLA−E
MZB1
FTL

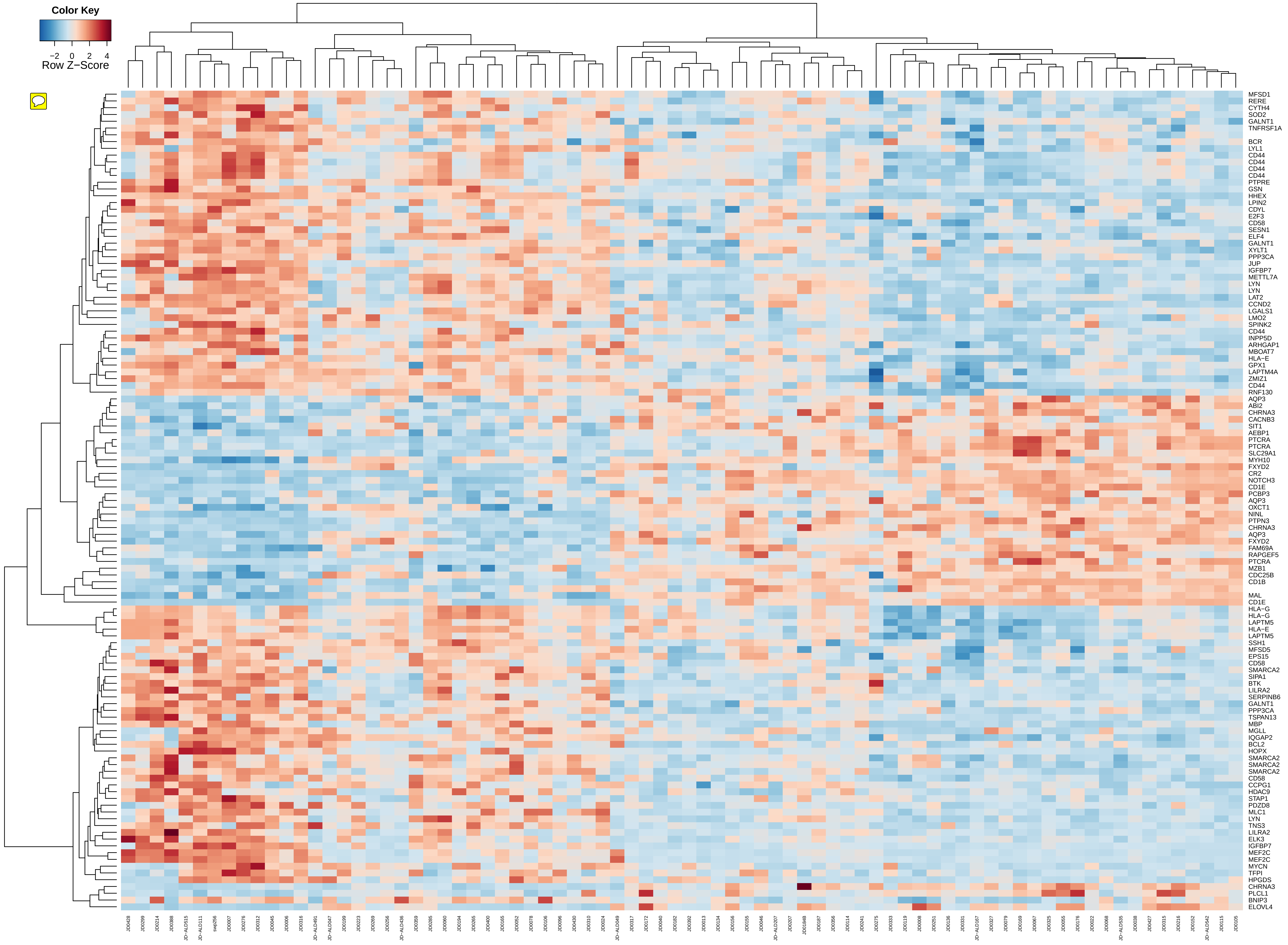
Color Key
−2 0 2 4
Row Z−Score
MFSD1
RERE
CYTH4
SOD2
GALNT1
TNFRSF1A
BCR
LYL1
CD44
CD44
CD44
CD44
PTPRE
GSN
HHEX
LPIN2
CDYL
E2F3
CD58
SESN1
ELF4
GALNT1
XYLT1
PPP3CA
JUP
IGFBP7
METTL7A
LYN
LYN
LAT2
CCND2
LGALS1
LMO2
SPINK2
CD44
INPP5D
ARHGAP1
MBOAT7
HLA−E
GPX1
LAPTM4A
ZMIZ1
CD44
RNF130
AQP3
ABI2
CHRNA3
CACNB3
SIT1
AEBP1
PTCRA
PTCRA
SLC29A1
MYH10
FXYD2
CR2
NOTCH3
CD1E
PCBP3
AQP3
OXCT1
NINL
PTPN3
CHRNA3
AQP3
FXYD2
FAM69A
RAPGEF5
PTCRA
MZB1
CDC25B
CD1B
MAL
CD1E
HLA−G
HLA−G
LAPTM5
HLA−E
LAPTM5
SSH1
MFSD5
EPS15
CD58
SMARCA2
SIPA1
BTK
LILRA2
SERPINB6
GALNT1
PPP3CA
TSPAN13
MBP
MGLL
IQGAP2
BCL2
HOPX
SMARCA2
SMARCA2
SMARCA2
CD58
CCPG1
HDAC9
STAP1
PDZD8
MLC1
LYN
TNS3
LILRA2
ELK3
IGFBP7
MEF2C
MEF2C
MYCN
TFPI
HPGDS
CHRNA3
PLCL1
BNIP3
ELOVL4
JD0428
JD0299
JD0214
JD0388
JD−ALD515
JD−ALD111
swp256
JD0007
JD0276
JD0312
JD0045
JD0006
JD0316
JD−ALD491
JD−ALD047
JD0199
JD0223
JD0269
JD0256
JD−ALD436
JD0359
JD0285
JD0060
JD0194
JD0265
JD0400
JD0165
JD0052
JD0078
JD0106
JD0096
JD0430
JD0310
JD0024
JD−ALD049
JD0317
JD0172
JD0040
JD0182
JD0392
JD0013
JD0134
JD0156
JD0155
JD0046
JD−ALD207
JD0207
JD0184B
JD0187
JD0356
JD0114
JD0241
JD0275
JD0333
JD0119
JD0008
JD0251
JD0136
JD0331
JD−ALD167
JD0327
JD0079
JD0169
JD0067
JD0325
JD0055
JD0176
JD0022
JD0068
JD−ALD535
JD0038
JD0427
JD0315
JD0216
JD0152
JD−ALD542
JD0115
JD0105

Figure S3

A

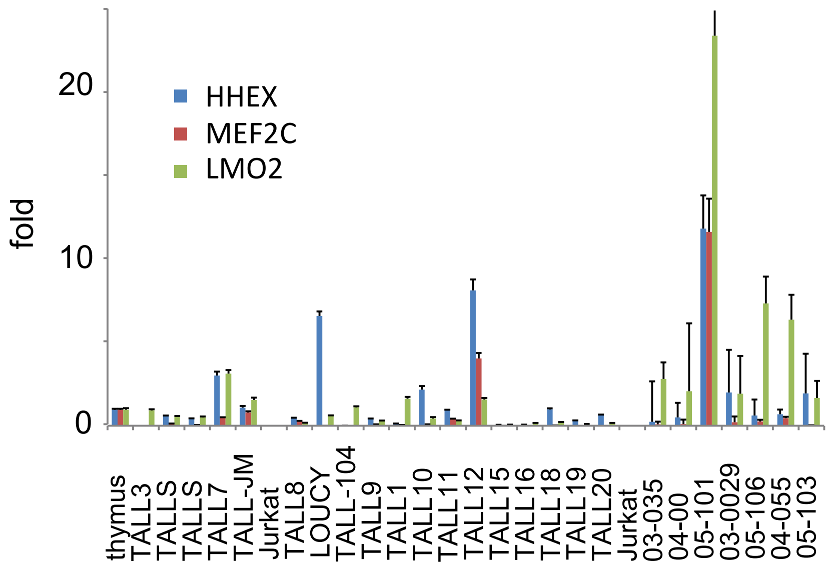

HHEX
MEF2C
LMO2
fold
20
10
0
thymus
TALL3
TALLS
TALLS
TALL7
TALL-JM
Jurkat
TALL8
LOUCY
TALL-104
TALL9
TALL1
TALL10
TALL11
TALL12
TALL15
TALL16
TALL18
TALL19
TALL20
Jurkat
03-035
04-00
05-101
03-0029
05-106
04-055
05-103

B

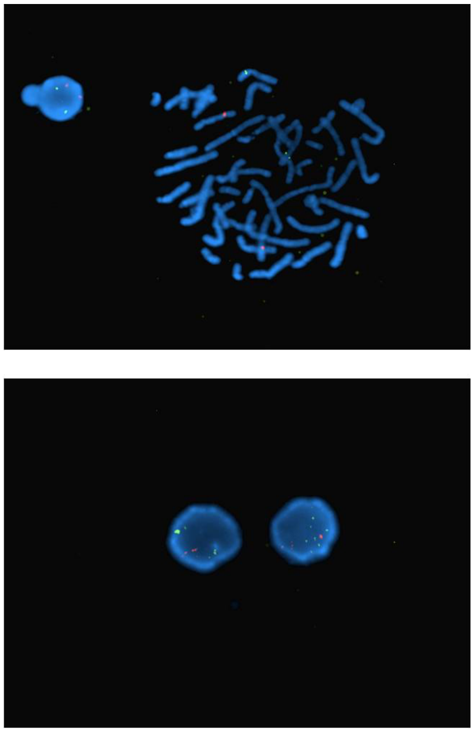

Figure S4

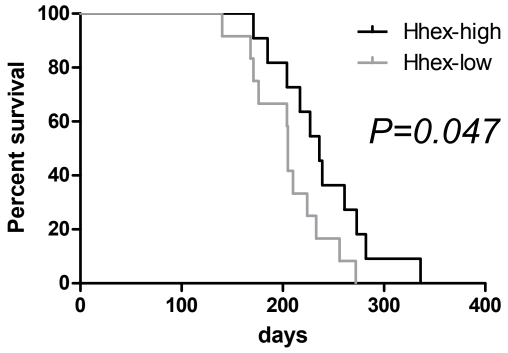
Percent survival
100
80
60
40
20
0
0
100
200
300
400
days
Hhex-high
Hhex-low
P=0.047
Percent survival
100
80
60
40
20
0
0
100
200
300
400
days
Dtx1-high
Dtx1-low
P=0.029

Figure S5

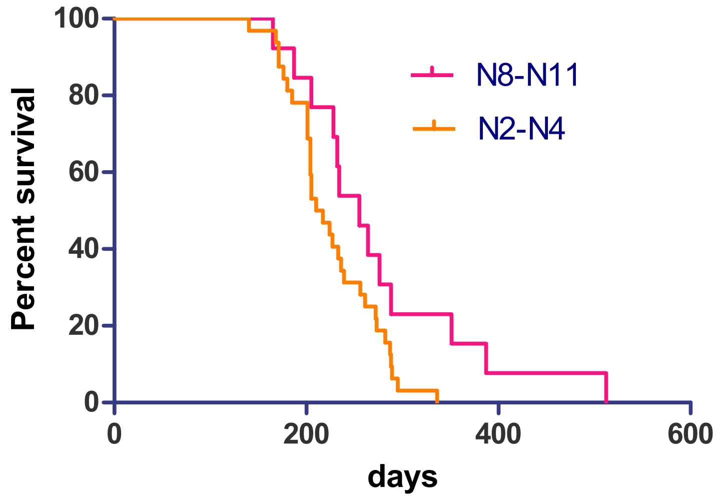

100
80
60
40
20
0
Percent survival
0
200
400
600
days
N8-N11
N2-N4

Figure S6

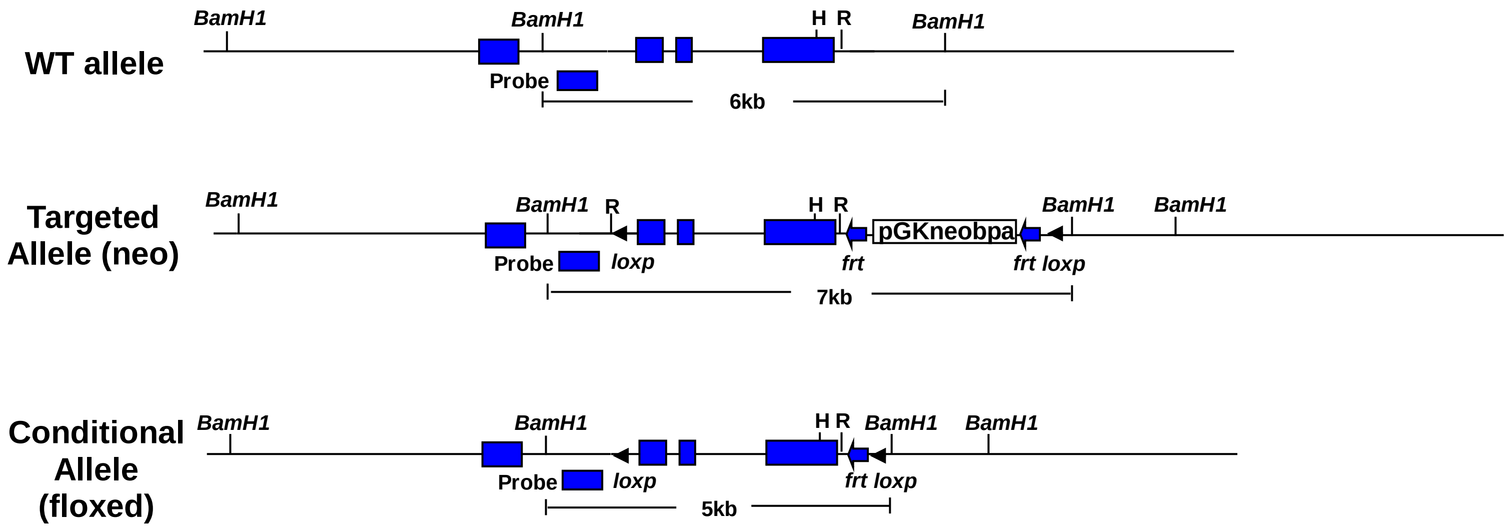

WT allele
BamH1
BamH1
H
R
BamH1
Probe
6kb
Targeted Allele (neo)
BamH1
BamH1
R
H
R
pGKneobpa
BamH1
BamH1
Probe
loxp
frt
frt loxp
7kb
Conditional Allele (floxed)
BamH1
BamH1
H
R
BamH1
BamH1
Probe
loxp
frt loxp
5kb

Figure S7

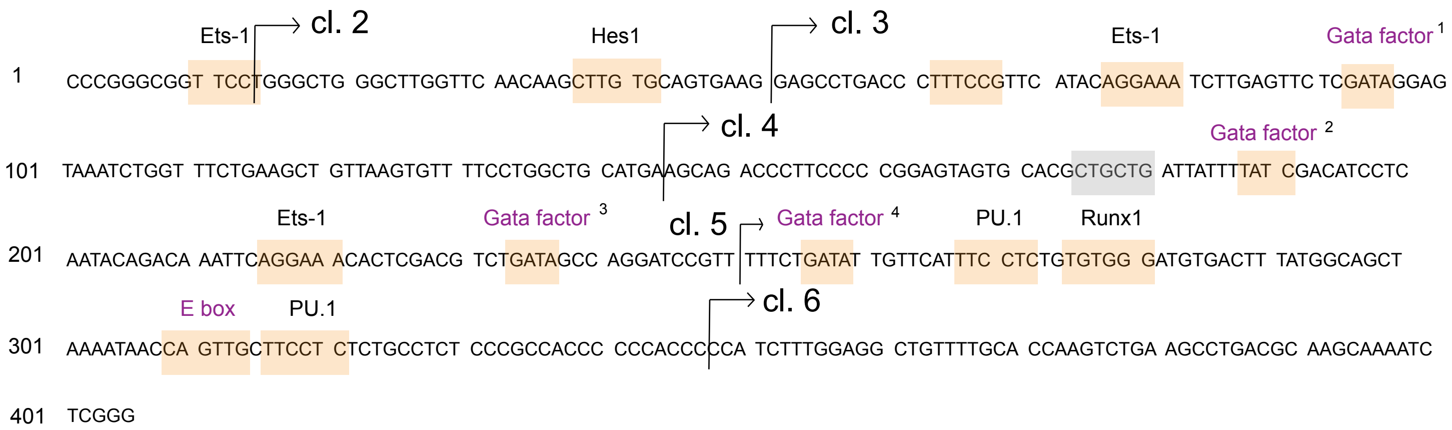

Ets-1
cl. 2
Hes1
cl. 3
Ets-1
Gata factor 1
1 CCCGGGCGGT TCCTGGGCTG GGCTTGGTTC AACAAGCTTG TGCAGTGAAG GAGCCTGACC CTTTCCGTTC ATACAGGAAA TCTTGAGTTC TCGATAGGAG
cl. 4
Gata factor 2
101 TAAATCTGGT TTCTGAAGCT GTTAAGTGTT TTCCTGGCTG CATGAAGCAG ACCCTTCCCC CGGAGTAGTG CACGCTGCTG ATTATTTTAT CGACATCCTC
Ets-1
Gata factor 3
cl. 5
Gata factor 4
PU.1
Runx1
201 AATACAGACA AATTCAGGAA ACACTCGACG TCTGATAGCC AGGATCCGTT TTTCTGATAT TGTTCATTTC CTCTGTGTGG GATGTGACTT TATGGCAGCT
E box
PU.1
cl. 6
301 AAAATAACCA GTTGCTTCCT CTCTGCCTCT CCCGCCACCC CCCACCCCCA TCTTTGGAGG CTGTTTTGCA CCAAGTCTGA AGCCTGACGC AAGCAAAATC
401 TCGGG

Figure S8

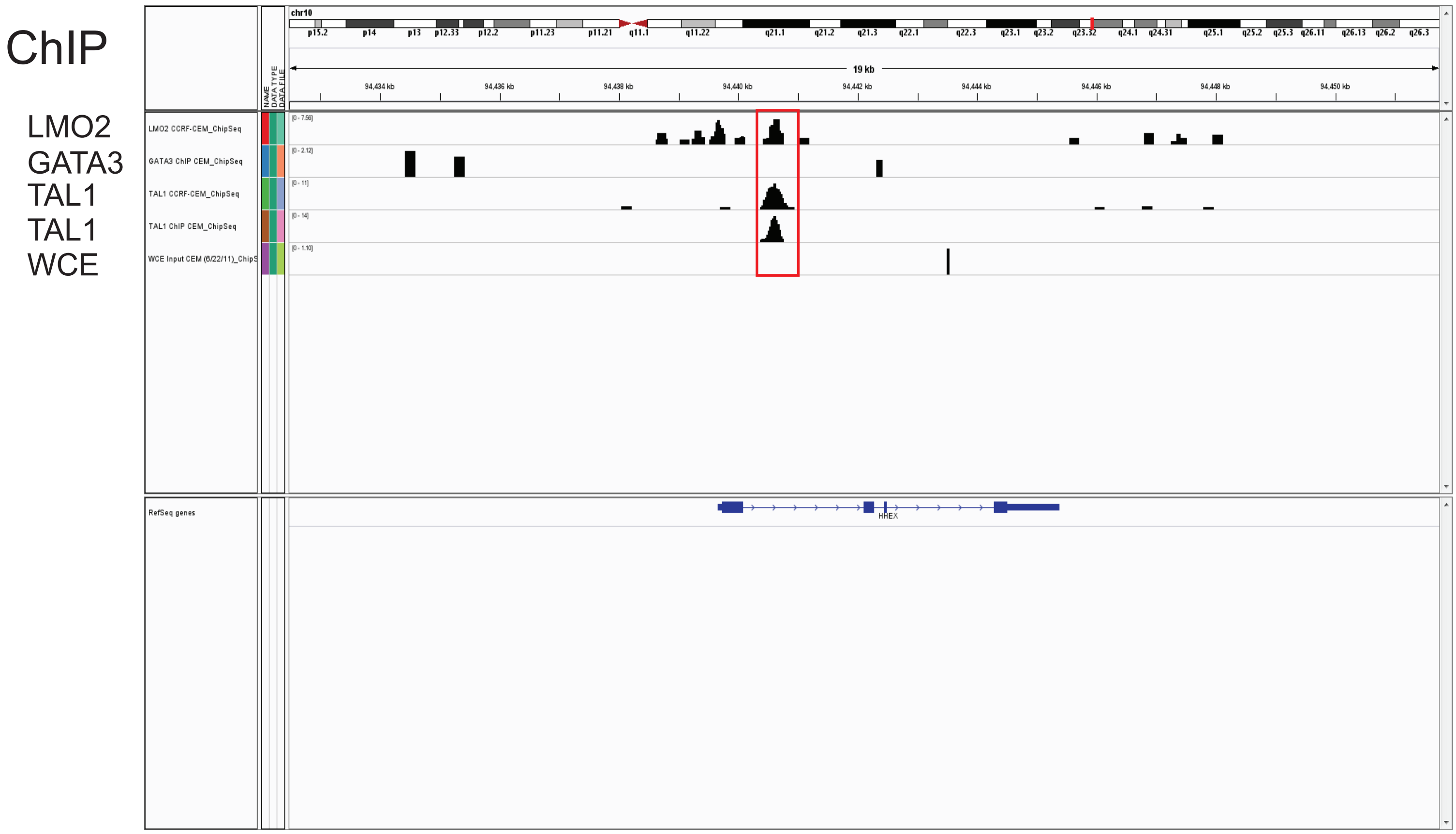
ChIP
LMO2
GATA3
TAL1
TAL1
WCE
chr10
19 kb
94,434 kb
94,436 kb
94,438 kb
94,440 kb
94,442 kb
94,444 kb
94,446 kb
94,448 kb
94,450 kb
LMO2 CCRF-CEM_ChipSeq
GATA3 ChIP CEM_ChipSeq
TAL1 CCRF-CEM_ChipSeq
TAL1 ChIP CEM_ChipSeq
RefSeq genes
HHEX

References

1. Irizarry RA, Hobbs B, Collin F, et al. Exploration, normalization, and summaries of high density oligonucleotide array probe level data. *Biostatistics (Oxford, England).* Apr 2003;4(2):249-264.

2. Smyth GK. Linear models and empirical bayes methods for assessing differential expression in microarray experiments. *Stat Appl Genet Mol Biol.* 2004;3:Article3.

3. Elliott NE, Cleveland S, Grann V, Janik JE, Dave UP. The Role of JAK3 mutations in Adult T-Cell Leukemia/Lymphoma. *ASH Annual Meeting Abstracts.* 2009;114(22):1940.

4. Weckx S, Del-Favero J, Rademakers R, et al. novoSNP, a novel computational tool for sequence variation discovery. *Genome Res.* Mar 2005;15(3):436-442.

5. Dignam JD, Lebovitz RM, Roeder RG. Accurate transcription initiation by RNA polymerase II in a soluble extract from isolated mammalian nuclei. *Nucleic Acids Res.* Mar 11 1983;11(5):1475-1489.

6. Cai Y, Xu Z, Xie J, et al. Eto2/MTG16 and MTGR1 are heteromeric corepressors of the TAL1/SCL transcription factor in murine erythroid progenitors. *Biochem Biophys Res Commun.* Dec 11 2009;390(2):295-301.

7. Liu P, Jenkins NA, Copeland NG. A highly efficient recombineering-based method for generating conditional knockout mutations. *Genome Res.* Mar 2003;13(3):476-484.

8. O'Neil J, Calvo J, McKenna K, et al. Activating Notch1 mutations in mouse models of T-ALL. *Blood.* Jan 15 2006;107(2):781-785.
